# A dual inhibitor of PIP5K1C and PIKfyve prevents SARS-CoV-2 entry into cells

**DOI:** 10.1038/s12276-024-01283-2

**Published:** 2024-08-01

**Authors:** Yuri Seo, Yejin Jang, Seon-gyeong Lee, Joon Ho Rhlee, Sukyeong Kong, Thi Tuyet Hanh Vo, Myung hun Kim, Myoung Kyu Lee, Byungil Kim, Sung You Hong, Meehyein Kim, Joo-Yong Lee, Kyungjae Myung

**Affiliations:** 1 Graduate School of Analytical Science and Technology, Chungnam National University, Daejeon, Republic of Korea; 2 Center for Genomic Integrity, Institute for Basic Science (IBS), Ulsan, Republic of Korea; 3 Infectious Diseases Therapeutic Research Center, Korea Research Institute of Chemical Technology (KRICT), Daejeon, Republic of Korea; 4 Department of Biological Science, Ulsan National Institute of Science and Technology, Ulsan, Republic of Korea; 5 Department of Chemistry, Ulsan National Institute of Science and Technology (UNIST), Ulsan, Republic of Korea; 6 Department of Biomedical Engineering, Ulsan National Institute of Science and Technology, Ulsan, Republic of Korea; 7 Graduate School of New Drug Discovery and Development, Chungnam National University, Daejeon, Republic of Korea; 8 Present Address: CasCure Therapeutics, Seoul, Republic of Korea

**Keywords:** Viral infection, Endosomes, Drug development

## Abstract

The SARS-CoV-2 pandemic has had an unprecedented impact on global public health and the economy. Although vaccines and antivirals have provided effective protection and treatment, the development of new small molecule-based antiviral candidates is imperative to improve clinical outcomes against SARS-CoV-2. In this study, we identified UNI418, a dual PIKfyve and PIP5K1C inhibitor, as a new chemical agent that inhibits SARS-CoV-2 entry into host cells. UNI418 inhibited the proteolytic activation of cathepsins, which is regulated by PIKfyve, resulting in the inhibition of cathepsin L-dependent proteolytic cleavage of the SARS-CoV-2 spike protein into its mature form, a critical step for viral endosomal escape. We also demonstrated that UNI418 prevented ACE2-mediated endocytosis of the virus via PIP5K1C inhibition. Our results identified PIKfyve and PIP5K1C as potential antiviral targets and UNI418 as a putative therapeutic compound against SARS-CoV-2.

## UNI418: Novel antiviral agent targeting SARS-CoV-2 entry and endosomal escape

The COVID-19 pandemic, triggered by the SARS-CoV-2 virus, underscores the immediate need for effective treatments, particularly for severe cases. Even with vaccines, treatments that block the virus’s entry into cells are vital. SARS-CoV-2 enters host cells by attaching to the ACE2 receptor, a process that is a prime target for intervention. This research concentrates on blocking the virus’s entry into cells as a potential treatment method. The study is an experiment using cellular models to assess the effectiveness of a new compound, UNI418, in preventing SARS-CoV-2 infection. UNI418 targets enzymes involved in cell membrane dynamics, essential for the virus’s entry. The researchers conclude that UNI418, by blocking PIP5K1C and PIKfyve, offers a promising approach to preventing SARS-CoV-2 infection and emphasizes the importance of targeting the virus’s entry process as a treatment strategy. This summary was initially drafted using artificial intelligence, then revised and fact-checked by the author.

## Introduction

Severe acute respiratory syndrome coronavirus 2 (SARS-CoV-2) caused the coronavirus disease 2019 (COVID-19) pandemic, resulting in an unprecedented crisis in public health and the global economy. In addition to vaccines, to fully overcome the COVID-19 pandemic, the development of novel therapeutics against SARS-CoV-2, especially for severe cases of COVID-19, is essential to reduce mortality.

Since coronavirus entry into host cells is critical for viral infectivity and pathogenesis^1,2^, this step is considered a crucial target for therapeutic intervention^3^. During viral entry into host cells, SARS-CoV-2 binds to the host cellular receptor, angiotensin-converting enzyme 2 (ACE2), which was initially reported in 2003 as the receptor for SARS-CoV^4–7^. The entry steps of SARS-CoV-2, including viral attachment to host cells, endocytosis, and membrane fusion, rely on interactions involving the spike (S) glycoprotein and ACE2^4–7^.

The spike protein of SARS-CoV-2 is cleaved into the S1 and S2 subunits in virus-producing cells by proprotein convertases, such as furin^2,8^. Therefore, mature SARS-CoV-2 comprises two noncovalently associated S subunits: S1, which is responsible for binding to ACE2, and S2, which anchors the spike protein homotrimer complex to the cell membrane^9^. Notably, S2 contains a fusion peptide, which is a hydrophobic stretch of residues that inserts into target cell membranes, facilitating membrane fusion. Specifically, the S2 subunit has a cleavage site, termed the “S2′ site”, which is recognized by transmembrane protease serine 2 (TMPRSS2) on the cell surface^8,10^. This cleavage event leads to the formation of a fusion pore, enabling the direct release of the viral genome into the cytoplasm during infection^10–13^. Additionally, independent of TMPRSS2, full-length spike protein can be cleaved into the fusogenic S2 subunit by cathepsin L^13–15^ within the acidified endosomal compartment subsequent to clathirin-mediated endocytosis^16,17^. Therefore, each viral entry step has been highlighted as a potential therapeutic target for preventing SARS-CoV-2 infection.

Inositol phospholipids play pivotal regulatory roles in cellular physiology through interactions mediated by their headgroups, which can be reversibly phosphorylated by the concerted action of phosphoinositide (PI) kinases and phosphatases to control key biological processes, most notably endocytosis, membrane trafficking, cytoskeleton dynamics, and autophagy^18^. Among phosphoinositide kinases, the phosphoinositide kinase for five position containing a FYVE finger (PIKfyve) is a critical enzyme involved in vesicle trafficking that is essential for the viral life cycle. It has been highlighted as a promising therapeutic target for emerging viral diseases such as those caused by the Ebola and Marburg viruses^19–21^. Importantly, PIKfyve has recently been identified as a putative therapeutic target against SARS-CoV-2 using specific inhibitors (apilimod, YM201636 and SGC-PIKFYVE-1)^20,22,23^. In addition, phosphatidylinositol 4-phosphate 5-kinases (PIP5Ks, including isotypes PIP5K1A, PIP5K1B, and PIP5K1C) produce PtdIns(4,5)P_2_ on clathirin-coated vesicles^24^, which are required for SARS-CoV-2 cellular entry^16,17^. However, the potential impact of targeting PIP5K on SARS-CoV-2 endocytosis and entry into cells remains unknown.

Previously, we identified ML367 as a small molecule that suppresses DNA replication stress responses induced by 5-fluororuidine (5-FUrd)^25^. Derivatives of ML367, which possess better activity in terms of inhibiting DNA replication stress, were recovered among the small molecules having similar structures. Of approximately 100 derivatives, we found that UNI418 potently suppressed the growth of PARP inhibitor-resistant tumors.

In this study, we characterized UNI418, a novel PIP5K1C and PIKfyve dual inhibitor. We provide evidence that UNI418 can inhibit SARS-CoV-2 infection in cellular models and suggest that this class of PIPK inhibitors has the potential to be developed into effective antiviral agents against SARS-CoV-2.

## Materials and Methods

### Synthesis of compound UNI418

***N*** **-(5-phenyl-1** ***H*** **-pyrazol-3-yl)-2-(4-pyridinyl)-4-quinazolinamine** 
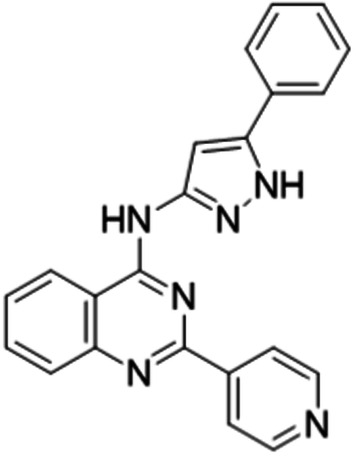


4-Chloro-2-(4-pyridyl)-quinazoline (0.83 mmol, 200 mg) and 3-amino-5-phenyl-*1H*-pyrazole (0.87 mmol, 139 mg) were dissolved in DMF. The reaction mixture was stirred for 12 hours at 80 °C. The solvent was evaporated, and the mixture was washed with EtOAc (100 mL × 3) and brine (200 mL × 3). The organic layer was dried and dissolved in EtOH (20 mL) and heated to reflux. After 15 minutes, the solution was cooled and allowed to stand overnight. Then, the solid was filtered to give 200 mg (66%) of brown powder. The powder was decolored with activated carbon (100 mg) and filtered to give a yellow powder (200 mg, 66%). ^1^H NMR (400 MHz, DMSO-*d*_6_) δ 13.12 (s, 1H), 10.70 (s, 1H), 8.79 (dd, *J* = 4.5, 1.6 Hz, 2H), 8.74 (d, *J* = 8.2 Hz, 1H), 8.35 (d, *J* = 5.7 Hz, 2H), 7.99–7.89 (m, 2H), 7.83 (t, *J* = 10.8 Hz, 2H), 7.64 (dd, *J* = 15.9, 9.7 Hz, 1H), 7.55 (dd, *J* = 16.1, 8.6 Hz, 2H), 7.46–7.33 (m, 2H). ^13^C NMR

### Cells

Vero81 (ATCC; CCL-81), Vero E6 (ATCC; CRL-1586), HCT116 (ATCC; CCL-247) and HEK293T (ATCC, CRL-11268) cells were maintained in DMEM supplemented with 10% fetal bovine serum (Life Technologies, Carlsbad, CA, USA), 100 U/mL penicillin G (Life Technologies) and 100 µg/mL streptomycin (Life Technologies). RPE1 cells were cultured in DMEM/F12 supplemented with 10% fetal bovine serum, 100 U/mL penicillin G and 100 µg/mL streptomycin. Stable HEK293T-hACE2 cells were provided by Prof. H-R Lee (Korea University) as a gift, and stable mCherry-GFP-LC3 HCT116 cells were generated.

### Chemicals

The following chemicals were purchased from the indicated companies: bafilomycin A_1_ (Cayman Chemical Company, Ann Arbor, MI, USA; 11038), dynasore (Sigma‒Aldrich, St. Louis, MO, USA; D7639), Hoechst (Cayman Chemical Company, 15547), UNC3230 (MedChem Express, NJ, USA; HY-110150), remdesivir (AdooQ, Irvine, CA, USA; A17170) and apilimod (MedChem Express, HY-14644). In particular, the purities of remdesivir and apilimod, which were used as antiviral controls, were confirmed to be greater than 95%.

### Cellular thermal shift assay (CETSA)

The chemically treated cells were heated to 56 °C for 3 min to denature and precipitate nontargeted proteins. The heated cells were immediately snap-frozen in liquid nitrogen overnight. After the cells were frozen and thawed twice, the tubes were briefly vortexed. The resulting cell lysates were subjected to SDS‒PAGE.

### mCherry-GFP-LC3 tandem fluorescent protein quenching assay

HEK293T cells at a density of 1.6 × 10^4^ cells per well in 96-well plates (Nunc) were stained with Hoechst (Cayman Chemical Company, 33342) for 10 min. The cells were washed with PBS two times and incubated with prewarmed media supplemented with 10% FBS. Cells were treated with UNI418 in prewarmed media. Images were acquired in a time-dependent manner and quantified with an ImageXpress high-content screening system (Molecular Devices, San Jose, CA, USA).

### Endosomal pH change test

This image-based experiment was performed according to our previous report with some modifications^26^. Vero cells were cultured in a glass bottom 35 mm dish (6 × 10^4^ cells per well) overnight. Each compound, including 1 µM UNC3230, apilimod or UNI418, was added to Vero cells for 1 h at 37 °C. The cells were stained with acridine orange (Sigma‒Aldrich, A9231) at a final concentration of 4 µg/mL for 30 min. After washing with PBS twice, images were acquired using a confocal microscope (Zeiss LSM 700, Baden-Württemberg, Germany) at two emission wavelengths, 493 to 560 nm and 590 to 720 nm, with excitation at 488 nm. The green-to-red ratio or vacuole size was measured using the built-in software ZEN 3.1.

### SARS-CoV-2 purification

SARS-CoV-2 (hCoV-19/Korea/KCDC06/2020), which belongs to clade S, was obtained from the Korea Disease Control and Prevention Agency and amplified in Vero E6 cells (passage no. 4). The virus was concentrated for use in infections at a high multiplicity of infection (MOI) according to a previous report^27^. Briefly, a virus stock (30 mL) was loaded onto 6 ml of 20% sucrose and subjected to ultracentrifugation using an SW-32Ti rotor (Beckman Instruments, Brea, CA, USA) at 106,800 × g for 16 h at 4 °C. After removal of the supernatant, the pellet was dissolved in 1.5 ml of DMEM overnight at 4 °C. The virus was titered by plaque assay in Vero E6 cells and stored at −80 °C before use. All experiments with infectious SARS-CoV-2 were performed in a biosafety level 3 (BL3) facility at KRICT.

### Preparation of SARS-CoV-2 spike protein pseudotyped lentivirus

To generate SARS-CoV-2 spike-pseudotyped lentivirus with luciferase, 5 × 10^6^ HEK293FT cells were cotransfected with 6 μg of pBOBI-Fluc, 4.5 μg of psPAX2 and 1.5 μg of SARS-CoV-2 spike plasmid (pcDNA3.1-SARS2-Spike for Wuhan-Hu-1, pcDNA3.3-SARS2-B1.617.2 for the Delta variant and pcDNA3.3_SARS2_omicron_BA.1 for the Omicron variant) using branched PEI (Sigma‒Aldrich). After 24 hours, the medium was removed from the cells, and fresh medium was added. The viral supernatants were harvested at 48 h and 72 h after transfection. pMD2.G was cotransfected with all the plasmids described above to package the VSV-G pseudovirus. For the recombinant SARS-CoV-2 spike protein, 5 × 10^6^ HEK293T cells were transfected with 12 μg of the SARS-CoV-2 HexaPro plasmid using branched PEI. After 24 h, the supernatant was removed, the medium was replaced with fresh medium, and the cells were harvested via the same procedure.

### Viral entry assay using a SARS-CoV-2 spike-pseudotyped virus with a luciferase reporter

A total of 1.5 × 10^4^ 293T-ACE2 cells were seeded in 96-well plates. Before virus infection (100 μL/each well), the cells were pretreated with 0.5 μM UNI418 for 1 h. After 12 h of infection, the supernatant was removed, and the medium was replaced with fresh medium. Luciferase activities were determined by a One-Glo Ex Luciferase Assay system (Promega Corporation, Madison, WI, USA; E8110) and analyzed with cell entry efficiency by GloMax (Promega Corporation) at 24 h postinfection.

### Cytotoxicity and antiviral assays

To assess the cytotoxicity and antiviral activity of compounds, including UNI418, apilimod, UNC3230 and remdesivir, Vero cells seeded on 96-well plates at a density of 3 × 10^4^ cells/well were mock-infected or infected with SARS-CoV-2 at an MOI of 0.01 (for UNC3230) or 0.001 (for UNI418, apilimod and remdesivir) in the presence of increasing concentrations of each compound (from 0.005 to 100 µM). Two days after incubation at 37 °C, mock-infected cells were lysed for a cell viability test using 3-(4,5-dimethylthiazol-2-yl)-2,5-diphenyltetrazolium bromide (MTT)^28^ at a final concentration of 2.5 mg/mL for 3 h at 37 °C. Viability was quantified by measuring the optical absorbance at 540 nm with a reference wavelength of 690 nm. The half maximal cytotoxic concentration (CC_50_) was defined as the concentration at which the compound reduced cell viability to 50% of that of 0.2% DMSO-treated cells. In parallel, virus-infected cells were fixed and permeabilized with chilled acetone-methanol solution (at a 1:3 ratio) for 10 min at room temperature. The viral spike protein was probed with an anti-spike antibody (GeneTex, Irvine, CA, USA) and Alexa 488-conjugated goat anti-mouse IgG (Invitrogen, A-11001), and the nuclei were counterstained with 4’,6-diamidino-2-phenylindole (DAPI; Vector Laboratories, Newark CA, USA; H-1200-10). Fluorescent cells were counted using an Operetta High-content Screening System (Perkin Elmer, Waltham, MA, USA). The half maximal effective concentration (EC_50_) was determined by measuring the concentrations of the compounds required for reducing the number of spike-positive cells by 50% relative to that of the virus-infected, 0.2% DMSO-treated cells. The antiviral activity and cytotoxicity of UNC3230 were measured on Day 1 due to its severe cytotoxicity on Day 2. For combination therapy, Vero cells infected with SARS-CoV-2 at an MOI of 0.01 were treated with 3-fold serial dilutions of different ratios of UNC3230 and apilimod: 5:0, 300 µM UNC3230 only; 4:1, 240 µM UNC3230 and 60 nM apilimod; 3:2, 180 µM UNC3230 and 120 nM apilimod; 2:3, 120 µM UNC3230 and 180 nM apilimod; 1:4, 60 µM UNC3230 and 240 nM apilimod; and 0:5, 300 nM apilimod only. On Day 1 after infection, the fractional EC_50_ (FEC_50_) values of UNC3230 and apilimod were individually determined at each fixed ratio.

### Visualization of the intracellular localization of NP and EEA1

Vero cells were cultured in 4-well chamber slides overnight (6 × 10^4^ cells per well). The cells were pretreated with 0.002% DMSO (mock), 1 µM UNI418 or 1 µM apilimod for 1 h at 37 °C. To synchronize virus infection, the culture medium was removed, and the cells were infected with highly purified SARS-CoV-2 at an MOI of 10 at 4 °C for 30 min in the presence of DMSO or each compound. Unadsorbed virus was removed by washing twice with PBS, and the samples were incubated at 37 °C in the same conditions with the compounds. At 1, 2, 4 and 8 h postinfection, the cells were fixed with 4% paraformaldehyde for 10 min and permeabilized with 0.1% Triton X-100 for 5 min. For fluorescence microscopy, viral nucleocapsid protein (NP) was labeled with a mouse anti-NP antibody (Sino Biological, China) and Alexa 488-conjugated goat anti-mouse IgG (Invitrogen), while cellular EEA1 was probed with a rabbit polyclonal anti-EEA1 antibody (Santa Cruz Biotechnology, Dallas, Texas, USA) and Alexa 633-conjugated goat anti-rabbit IgG. Nuclei in all samples were counterstained with DAPI. Images were captured using a confocal microscope (Zeiss LSM 700).

### Spike-RBD domain-induced receptor translocation

HEK293T cells were seeded on 35 mm confocal dishes (1.0 × 10^6^ cells) and transfected with 0.5 μg of ACE2-GFP using branched PEI. After 24 h, ACE2-transfected 293 T cells were pretreated with 0.1 mM dynasore, 0.5 μM UNC3230, apilimod or UNI418 in the presence of 20 μg/mL cycloheximide (Sigma Aldrich, C7698) for 30 min at 37 °C. The cells were then treated for the indicated times with 20 μg/mL spike-RBD protein together with UNC3230, apilimod or UNI418 at the same concentrations in the presence of cycloheximide. Images were acquired with a confocal microscope (Zeiss LSM 880 with Airyscan; Carl Zeiss) at each time point. The spike-RBD protein, a gift from Ho Min Kim (Institute for Basic Science), was purified.

## Results

### UNI418 is a specific inhibitor of PIKfyve and PIP5K1

**Fig. 1:**
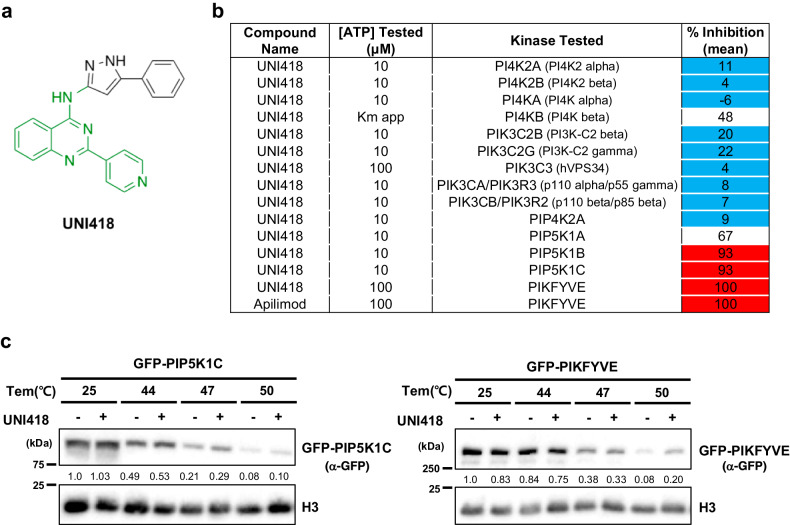
UNI418 specifically targets PIP5K1 and PIKfyve. **a** Chemical structure of UNI418. The 4-quinazolinamine core is highlighted in green. **b** Table of the kinase profiling analysis. Inhibition of several kinases was determined at a 1 μM concentration of UNI418 and analyzed by the SelectScreen Kinase Profiling Service (Thermo Fisher Scientific). **c** Cellular thermal shift assay. Ectopically expressed GFP-PIP5K1C and GFP-PIKfyve were heated to the indicated temperatures with/without UNI418, and the presence of protein in the soluble fraction was analyzed by Western blot analysis.

UNI418 has a core structure consisting of 4-quinazolinamine (Fig. 1a, green), which is often found in kinase inhibitors^29^. UNI418 treatment induced vacuolization (Supplementary Fig. 1), indicating that it plays an important role in cellular membrane trafficking. The phosphoinositide (PI) system is a crucial regulatory mechanism in membrane trafficking and is tightly regulated through phosphorylation cascades by multiple phosphatidylinositol phosphate kinases (PIPKs)^30^. We hypothesized that UNI418 may inhibit PIPK(s) and performed PIPK profiling analysis. UNI418 inhibited PIP5K1B (93% inhibition at 1 µM and IC_50_: 60.7 nM), PIP5K1C (93% inhibition at 1 µM, IC_50_: 60.1 nM and K_d_: 61 nM), and PIKfyve (100% inhibition at 1 µM and K_d_: 0.78 nM) and partially inhibited PIP5K1A (67% inhibition at 1 µM and IC_50_: 255 nM), indicating that UNI418 primarily targets PIKfyve, with PIP5K1B/C as secondary targets (Fig. 1b and Supplementary Fig. 2). To test whether UNI418 interacts with PIP5K1C and PIKfyve, we performed a CETSA, which determines the characteristics of protein stabilization upon ligand binding to the target protein^31^. UNI418- or DMSO-treated cell lysates were incubated at different temperatures, and the presence of the target protein in the soluble fraction was analyzed by Western blotting (Fig. 1c). When UNI418 was applied, exogenously expressed PIP5K1C and PIKfyve became more stable at higher temperatures than did the DMSO control, indicating that UNI418 interacts with PIP5K1C and PIKfyve in cells (Fig. 1c). We also demonstrated that UNI418 treatment reduced the levels of PtdIns(4,5)P_2_ (Supplementary Fig. 3), which is synthesized mainly by PIP5Ks^32,33^.

Using specific inhibitors of PIKfyve (apilimod) and PIP5K1C (UNC3230)^34,35^, UNI418-induced vacuoles were found to be positive for EEA1 (an early endosome marker) and LAMP1 (a lysosomal marker), similar to apilimod-induced vacuoles. In contrast, UNC3230 did not induce vacuoles, indicating that PIKfyve was responsible for UNI418-induced vacuolization (Supplementary Figs. 4 and 5). Both UNI418- and apilimod-induced vacuoles gradually disappeared over time after washout, indicating that both compounds acted reversibly (Supplementary Fig. 5).

### UNI418 inhibits autophagy

**Fig. 2:**
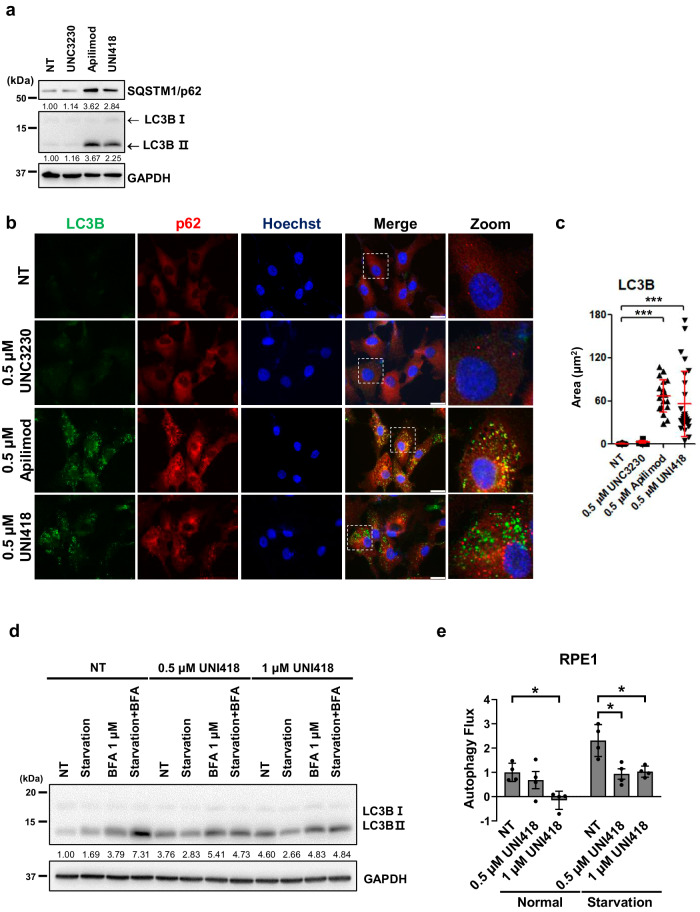
UNI418 inhibits autophagy. **a** Western blot analysis of p62, LC3B-I, LC3B-II and GAPDH in the presence of UNC3230, apilimod and UNI418. **b** Immunofluorescence staining with LC3B (green) and p62 (red) antibodies upon treatment with UNC3230, apilimod and UNI418, and images within the white rectangular boxes are magnified on the right side. Scale bar, 20 µm. **c** The area of fluorescence intensity from immunofluorescence images (**b**) was quantified after UNC3230, apilimod and UNI418 treatments. **d, e** Quantification of autophagic flux under normal conditions and under HBSS-induced starvation conditions with or without UNI418 treatment. RPE1 cells were treated with UNI418 (0.5 µM or 1 µM) for 2 h in the presence or absence of a lysosomal inhibitor (bafilomycin A_1_, BFA). Representative Western blot images (**d**) and the graph showing the average of 4 independent experiments (**e**). The error bars indicate the S.D. Statistical analysis was performed using two-way ANOVA; *p < 0.05, ***p < 0.001.

Since UNI418 specifically inhibits PIP5K1B, PIP5K1C, and PIKfyve, which have been reported to be crucial regulators of trafficking to lysosomes and autophagosome-lysosome fusion^36,37^, we examined whether UNI418 inhibited autophagy using known specific inhibitors of PIKfyve and PIP5K1C. Treatment of RPE1 cells with UNI418 and apilimod strongly induced the accumulation of two autophagy-monitoring proteins, SQSTM1/p62 (sequestosome 1), a typical substrate for autophagy^38^, and LC3-II, a widely accepted marker for autophagolysosomal turnover^39^, while UNC3230 did not have the same effect (Fig. 2a). Consistently, immunofluorescence staining for SQSTM1/p62 (red) and LC3 (green) revealed a significant accumulation of LC3-positive and SQSTM1/p62-positive puncta in UNI418- or apilimod-treated RPE1 cells but not in UNC3230-treated RPE1 cells (Fig. 2b, c). Since autophagic flux has been defined as autophagic degradation activity, we examined whether UNI418 treatment blocked autophagic flux by measuring the turnover rate of LC3-II^39^. To measure autophagic flux, lysosomal degradation was inhibited by bafilomycin A_1_ (BFA). In normal growth media, treatment with 1 μM UNI418 markedly inhibited autophagic flux (Fig. 2d, e). The inhibitory effect of UNI418 on autophagic flux was substantial after cell starvation in HBSS containing D-glucose and essential inorganic ions, which lacks amino acids and serum and is commonly used for the study of starvation-induced autophagy. The increase in autophagic flux induced by HBSS in RPE1 cells was markedly inhibited by UNI418 treatment (Fig. 2d, e and Supplementary Fig. 6).

### UNI418 inhibits autophagosome-lysosome fusion and endolysosomal acidification

**Fig. 3:**
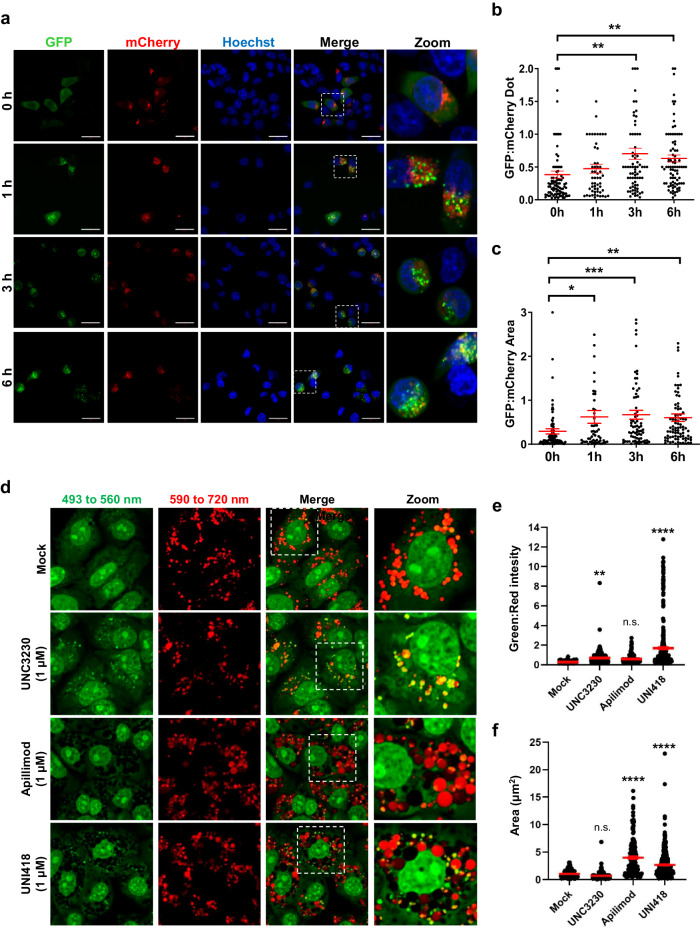
UNI418 negatively affects autophagosome-lysosome fusion and endosome acidification. **a** Live-cell imaging of HEK293T cells stably expressing mCherry-GFP-LC3 treated with 0.5 μM UNI418 at the indicated time points; the images within the white rectangular boxes are magnified on the right side. Scale bar, 30 µm. The number (**b**) and area (**c**) of GFP-positive dots per cell were quantified by ImageXpress. **d** Immunofluorescence images illustrating the inhibition of endolysosomal acidification by UNI418. Vero cells were treated with the mock control or 1 µM UNC3230, apilimod or UNI418 for 1 h at 37 °C. Acridine orange (4 µg/mL) was added to the compound-treated live cells, followed by additional incubation for 30 min. The cells were analyzed by confocal microscopy using two emission wavelengths at 493–560 (green) and 590–720 nm (red) after excitation at 488 nm. Merged images are presented, and images within the white rectangular boxes are magnified on the right side. Original magnification, 630×. The green-to-red fluorescence intensity ratio (**e**) and the area of fluorescent spots (**f**) were quantified. **b**, **c**, **e**, **f** The data are presented as the means ± SEMs, with a minimum of 200 spots. Statistical significance was determined by Student’s t test; *p < 0.05, **p < 0.01, ***p < 0.001 and ****p < 0.0001. n.s., not significant.

PIP5Ks and PIKfyve kinase are closely linked to autophagosome-lysosome fusion and endolysosomal acidification^40^. To investigate the role of UNI418 treatment in autophagosome-lysosome fusion, we used a pH-sensitive tandem-labeled mCherry-GFP-LC3 reporter to determine the efficiency of autophagosome-lysosome fusion in individual cells. Since GFP, but not mCherry, fluorescence is lost in acidic compartments, mCherry-GFP-LC3 labels nonacidic autophagosomes with yellow fluorescence (positive for both green and red) and acidic autolysosomes with red fluorescence only^41^. Notably, 0.5 μM UNI418 treatment considerably increased the number of nonacidic autophagosomes within 6 h (Fig. 3a–c), suggesting that UNI418 treatment inhibited autophagosome-lysosome fusion or lysosomal acidification.

To monitor endolysosomal acidification after UNI418 treatment, acridine orange, a cell-permeable pH-sensitive metachromatic dye, was used. After excitation at 488 nm, it emits green fluorescence (monitored at 560 nm) when it exists as a monomer under high pH conditions, whereas it produces red fluorescence (monitored at 720 nm) when it forms dimers under low pH conditions^42^. Thus, the emission of green fluorescence highlights high-pH vesicles, and the emission of red fluorescence highlights low-pH vesicles^43^. Vero cells were stained with acridine orange to monitor changes in vacuolar pH after UNI418 treatment. Furthermore, apilimod and UNC3230 were used as controls for discriminating PIKfyve and PIP5K1C inhibitory effects. In mock-treated cells, mostly red-positive puncta (low-pH vesicles) were observed (Fig. 3d, first row). Similarly to UNC3230 treatment, UNI418 treatment considerably increased the number of green-positive puncta (high-pH vesicles) (Fig. 3d, fourth row and second row). Quantitatively, the green-to-red (green:red) ratio of fluorescent spots was 2.6-fold greater in the UNC3230-treated sample and 6.3-fold greater in the UNI418-treated sample than in the mock-treated sample (Fig. 3e). Although the green/red ratio was 2.2 times greater in the apilimod-treated sample than in the mock-treated sample, there was no statistical significance (Fig. 3d, third row, and Fig. 3e). Interestingly, the increase in the number of intracellular vacuoles induced by apilimod was also induced by UNI418 (Fig. 3f). Collectively, these results suggested that UNI418 is a bifunctional molecule that targets both PIP5K1C and PIKfyve, thus inhibiting endolysosomal acidification, similar to UNC3230, and inducing vacuolization, similar to apilimod. This conclusion was further supported by similar results obtained using PIKfyve- and PIP5K1C-specific siRNA transfection (Supplementary Fig. 7).

To distinguish deacidification in endosomes and lysosomes, we performed the same experiment with an early/late endosomal marker (mCherry-Rab5/Rab7) and a lysosomal marker (mCherry-LAMP1). As shown in Supplementary Fig. 8, we found that deacidification by UNI418 occurs in Rab7-positive late endosomes and in LAMP1-positive lysosomes, with a minor fraction occurring within Rab5-positive early endosomes, potentially indicative of transitional endosomes from early to late stages.

### UNI418 inhibits cathepsin maturation by preventing PIKfyve-dependent lysosomal trafficking, which is relevant to SARS-CoV-2 spike protein cleavage

**Fig. 4:**
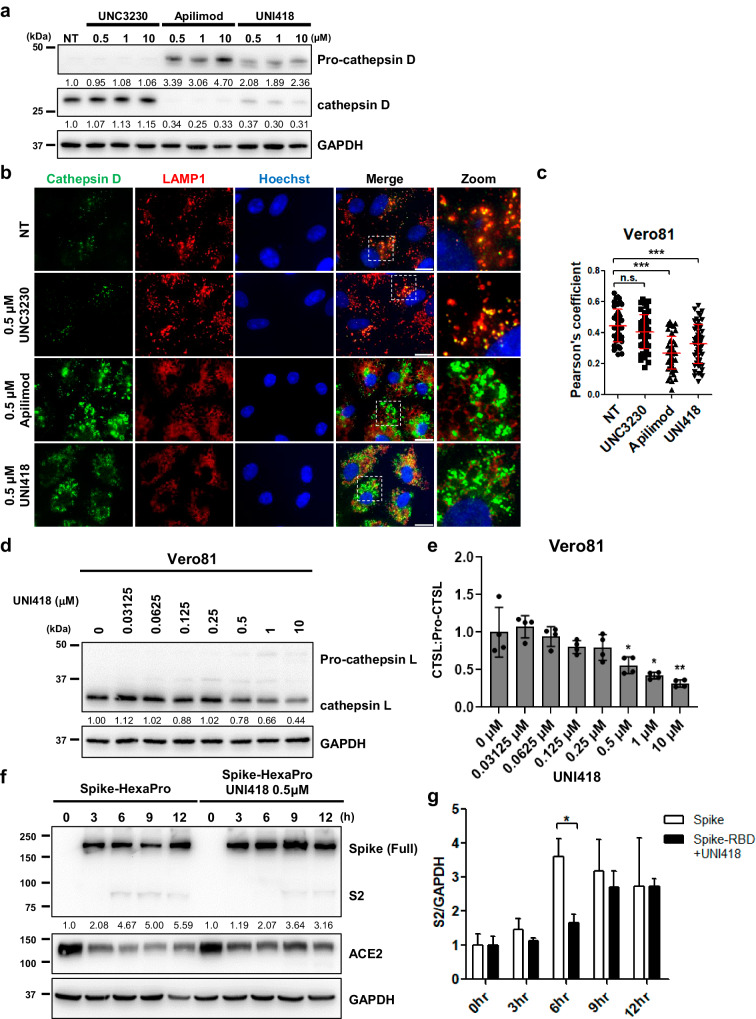
UNI418 delays the maturation of cathepsins and cleavage of the SARS-CoV-2 spike protein. **a** Representative Western blot image depicting pro- and mature cathepsin D after treatment with the indicated concentrations of UNC3230, apilimod and UNI418. GAPDH served as a loading control. **b**, **c** Evaluation of cathepsin D and LAMP1 colocalization in Vero cells after treatment with UNC3230, apilimod or UNI418 for 4 h. Immunofluorescence staining (**b**) and Pearson’s correlation coefficients (**c**) between LAMP1 and cathepsin D distribution. Scale bar, 15 µm. Images of individual cells, comprising at least 45 cells per sample, were randomly cropped and analyzed for colocalization using JACoP in ImageJ. The error bars indicate the SDs. Statistical significance was determined using Student’s t-test, where ***p < 0.001 and n.s., not significant. **d**, **e** Cathepsin L maturation in Vero cells was determined by Western blotting. **d** Representative Western blot images showing pro- and mature cathepsin L levels after treatment with UNI418 at the indicated concentrations. GAPDH was used as a loading control. **e** The ratio of cathepsin L to pro-cathepsin L was quantified and is presented as the mean ± SEM, with a sample size of *n* = 3. Statistical significance was determined by Student’s t test, where *p < 0.05 and **p < 0.01. **f** Cleavage of the SARS-CoV-2 spike protein was determined with or without UNI418 treatment at specific time points. HEK293T cells stably expressing ACE2 (293T-ACE2) were treated with SARS-CoV-2 spike protein (Spike-HexaPro) in the absence or presence of 0.5 μM UNI418 and subjected to Western blotting for the SARS-CoV-2 spike protein, ACE2 and GAPDH. **g** The ratios of processed S2/GAPDH were quantified and are presented as the means ± SEMs, with *n* = 4. Statistical analysis was performed using two-way ANOVA; *p < 0.05.

Given that UNI418 treatment inhibits endolysosomal acidification (Fig. 3d, e and Supplementary Fig. 8), we hypothesized that UNI418 treatment would inhibit the acidic pH-dependent maturation of cathepsins within endosomes or lysosomes. To validate this hypothesis, we investigated whether UNI418 treatment inhibited the proteolytic cleavage of cathepsin D with well-known specific inhibitors of PIKfyve and PIP5K1C. Unlike the vesicular acidification results (Fig. 3d, e), the results of this experiment showed that apilimod largely inhibited the proteolytic cleavage of cathepsin D, but UNC3230 did not (Fig. 4a). This result indicated that UNI418 inhibited proteolytic cathepsin maturation through PIKfyve rather than PIP5K1C-dependent vesicle acidification. To investigate other possible mechanisms, we analyzed whether cathepsin was properly localized within lysosomes by conducting a colocalization assay probing for cathepsin D and LAMP1. As shown in Fig. 4b, c, similar to apilimod treatment, UNI418 treatment largely reduced the lysosomal targeting of cathepsin D, which could be coupled with its nonlysosomal accumulation. However, UNC3230 did not have the same effect, indicating that PIKfyve was responsible for this effect. To avoid the possibility that UNI418 is a simple lysosomotropic reagent, we examined the accumulation of LAMP1 and LAMP1-positive lysosomes after treatment with UNI418 or chloroquine (a well-known lysosomotropic drug). Unlike chloroquine, UNI418 did not cause the accumulation of LAMP1 or LAMP1-positive lysosomes, which is a characteristic phenomenon associated with lysosomotropic drugs such as chloroquine, suggesting that UNI418 is not a simple lysosomotropic agent (Supplementary Fig. 9). The inhibitory effect of UNI418 on the proteolytic cleavage of cathepsin D was reproducible in other cell lines (RPE1 and HCT116) (Supplementary Fig. 10), as well as on cathepsin L in Vero cells (Fig. 4d, e, and Supplementary Fig. 11), which is known to regulate SARS-CoV-2 infection^12–15^. Throughout all the experiments, UNI418 treatment considerably inhibited the proteolytic maturation of both cathepsin D and cathepsin L.

Endosomal escape of SARS-CoV-2 relies on proteolysis of the full-length spike protein into its mature form, which occurs at a minimum of two cleavage sites and is catalyzed by host cathepsin L within late endosomes/lysosomes^12–15^. These sites, termed CS-1 and CS-2, are positioned at the N-terminal domain of the S1 subunit and proximal to the furin-cleaving S1/S2 site, respectively^15^. To examine whether UNI418 inhibited the proteolysis of the SARS-CoV-2 spike protein, we used an ectodomain-only SARS-CoV-2 spike HexaPro mutant protein (Spike-HexaPro) that contains mutations at the furin cleavage site while preserving other cleavage sites recognized by cathepsin L. We incubated hACE2-overexpressing HEK293T cells (referred to as hACE2-293T cells) with the Spike-HexaPro protein alone or in the presence of 0.5 μM UNI418 and then subjected them to Western blot analysis at specific time points. Spike-HexaPro protein cleavage, presumably at the CS-2 site, was observed using an S2-specific antibody after 6 h of incubation in control DMSO-treated cells (Fig. 4f, g). In contrast, UNI418 treatment delayed the generation of the mature product by 3 h (after 9 h of incubation). It has been reported that SARS-CoV-2 infection occurs upon spike-ACE2 binding and subsequent membrane fusion, accompanied by ACE2 internalization and its downregulation on the cell surface^2^. We also observed that UNI418 treatment alleviated the downregulation of ACE2 triggered by the spike protein in the same lysates (Fig. 4f).

### UNI418 inhibits SARS-CoV-2 infection of Vero cells

**Fig. 5:**
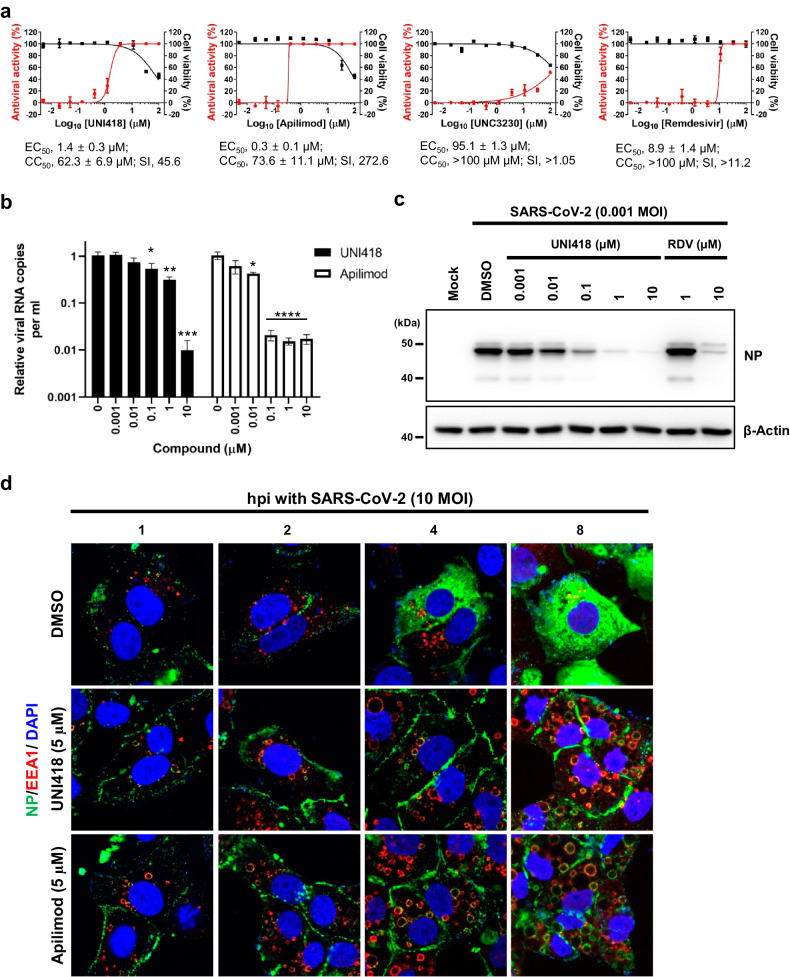
UNI418 protects against SARS-CoV-2 infection. **a** Antiviral activity and cytotoxicity of UNI418. Vero cells mock-infected or infected with SARS-CoV-2 at an MOI of 0.01 or 0.001 were individually treated with increasing concentrations of UNI418, with apilimod, UNC3230 and remdesivir used as controls. On Day 2 (for UNI418, apilimod and remdesivir) or Day 1 (for UNC3230) postinfection, the number of viral spike protein-positive cells was determined by immunofluorescence microscopy using an anti-spike antibody. The antiviral activity (EC_50_) was calculated as the concentration inhibiting the spike-positive cell number by 50% (red line). In parallel, the cytotoxicity (CC_50_) was determined as the concentration that reduced the viability of mock-infected cells by 50% using MTT (black line). The selectivity index (SI) is the ratio of the CC_50_ to the EC_50_. The values are expressed as the means ± SEMs from three independent experiments. **b** Reduction of SARS-CoV-2 genomic RNA as determined by quantitative RT‒PCR. Vero cells were infected with SARS-CoV-2 (MOI, 0.001) for 1 h at 37 °C. After removal of the unabsorbed virus, the cells were treated with increasing concentrations of UNI418 or apilimod for 24 h. Viral RNA was purified from culture supernatants and subjected to one-step quantitative RT‒PCR to measure relative amounts of the viral *NP* gene. The values are expressed as the means ± SEMs of three samples. Statistical significance was determined by two-way ANOVA with Dunnett’s multiple comparison test; *p < 0.05; ***p < 0.001; ****p < 0.0001. **c** Reduction in SARS-CoV-2 NP expression by UNI418. SARS-CoV-2-infected Vero cells were treated with increasing concentrations of UNI418 or remdesivir (RDV). On Day 1 postinfection, cell lysates were harvested to measure viral nucleocapsid protein (NP) expression by Western blot analysis. β-Actin was used as a loading control. The marker size is labeled on the left side of the gels. **d** Blockade of the endocytic pathway by UNI418 in SARS-CoV-2-infected cells. Vero cells were pretreated with DMSO as a mock control (top), 1 µM UNI418 (middle) or 1 µM apilimod (bottom) for 1 h at 37 °C. To synchronize viral infection, the cells were infected with highly purified SARS-CoV-2 (MOI, 10) for 1 h at 4 °C in the presence of each compound. After shifting to 37 °C again, the cells were fixed at 1, 2, 4, and 8 h postinfection for immunolabeling with antibodies specific for viral NP (green) and cellular EEA1 (red). Nuclei were counterstained with DAPI (blue) for all samples. Original magnification, 630×.

To test the antiviral efficacy of UNI418 against SARS-CoV-2 infection, Vero cells were infected with the virus and treated with increasing concentrations of UNI418, apilimod, UNC3230 and remdesivir were used as controls. On Day 2 (for UNI418, apilimod and remdesivir) or Day 1 (for UNC3230) postinfection, immunofluorescence staining analysis with an anti-spike antibody showed that viral infection was inhibited by all compounds in a dose-dependent manner (Fig. 5a and Supplementary Fig. 12). The EC_50_ value of UNI418 (1.4 µM) was between those of apilimod (0.3 µM) and remdesivir (8.9 µM). We also compared their cytotoxicity to determine each antiviral selectivity index (SI). MTT-based cell viability testing under the same cell culture conditions but without viral infection revealed CC_50_ values of 62.3 µM for UNI418 (SI calculated from the ratio of CC_50_ to EC_50_, 45.6), 73.6 µM for apilimod (SI, 272.6) and >100 µM for remdesivir (SI, >11.2). In contrast to the three active compounds, as UNC3230 was highly toxic on Day 2, we tested its inhibitory effect on Day 1. The results showed that UNC3230 was marginally active, with an EC_50_ of 95.1 µM and a CC_50_ of >100 µM. Overall, the antiviral and cytotoxicity data suggested that UNI418 was active against SARS-CoV-2 at subtoxic concentrations. To confirm its inhibitory effect, quantitative RT‒PCR and Western blot analyses were performed with SARS-CoV-2-infected culture supernatants and Vero cell lysates after treatment with UNI418 at different concentrations ranging from 1 nM to 10 µM. Titration of viral genome copies showed an approximately 2-log reduction in the levels of the *NP* gene by treatment with 10 µM UNI418 and apilimod (Fig. 5b). Consistent with this finding, the amount of viral NP drastically decreased as the concentration of UNI418 increased, which did not affect the expression levels of cellular β-actin (Fig. 5c). These data suggested that UNI418, an antiviral agent, efficiently inhibited SARS-CoV-2 replication in cells and reduced the production of progeny virus in culture supernatants.

We also explored whether endolysosomal deacidification and the inhibition of cathepsin L maturation by UNI418 (as shown in Figs. 3d, e and 4d, e) could influence the endosomal escape of SARS-CoV-2 at the entry step. For this study, virus-infected cells were incubated with two different primary antibodies specific for viral NP and cellular early endosome antigen 1 (EEA1), which were detected with Alexa 488- and Alexa 633-conjugated secondary antibodies, respectively (Fig. 5d and Supplementary Fig. 13a–d). A time-course immunofluorescence assay showed that in DMSO-treated mock cells, viral NP was colocalized with EEA1 around the perinuclear region at 1 h postinfection and became separated at a later time point (2 h postinfection). During one round of the viral life cycle, approximately 8 h^44^, the amount of newly synthesized viral NP was robustly increased in the cytoplasm (Fig. 5d, top panels and Supplementary Fig. 13e). In contrast, images of cells after UNI418 treatment revealed that UNI418 inhibited not only endosome-mediated SARS-CoV-2 entry by accumulating NPs on the plasma membrane but also the release of NPs from endosomal vesicles, the size of which increased gradually in a time-dependent manner (Fig. 5d, middle panels and Supplementary Fig. 13e). Interestingly, viral NP was localized along the EEA1-bound endosomal membrane. Similar results were observed in apilimod-treated cells (Fig. 5d, bottom panels and Supplementary Fig. 13e), as indicated in a recent report suggesting that inhibition of PIKfyve kinase not only induced abnormal enlargement and vacuolization of EEA1-positive endosomes with pseudotyped Ebolavirus but also blocked SARS-CoV-2 infection^20^.

**Fig. 6:**
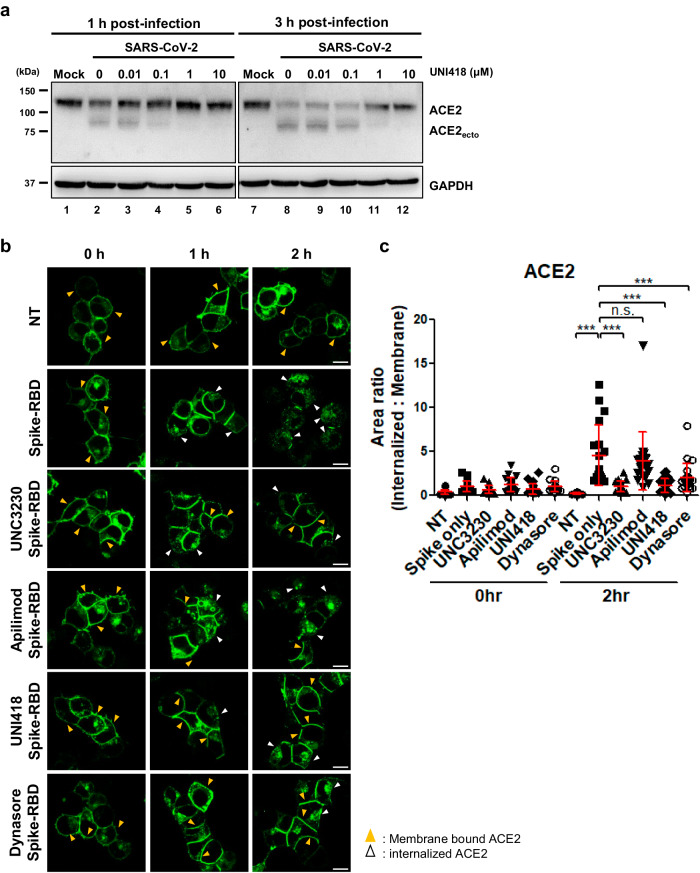
UNI418 inhibits ACE2 receptor-mediated viral entry. **a** SARS-CoV-2 infection-induced ACE2 downregulation was assessed in Vero cells. The cells were infected with SARS-CoV-2 at the indicated concentrations of UNI418 and subjected to Western blot analysis of ACE2, its cleaved ectodomain (ACE2_ecto_) and GAPDH at 1 and 3 h postinfection. **b** Endocytosis of ACE2 triggered by the interaction of ACE2 with the spike RBD protein (spike-RBD) was examined using 0.1 mM dynasore and 0.5 μM UNC3230, apilimod and UNI418. HEK293T cells were transfected with hACE2-GFP, treated with each compound and subjected to live cell imaging at the indicated times to visualize the hACE-GFP distribution. Membrane-bound ACE2 and internalized ACE2 are marked with yellow and white arrows, respectively. Scale bar, 10 µm. **c** The area ratio of internalized to membranous hACE2-GFP in individual cells was quantified and is presented as the mean ± SEM, *n* > = 20. Statistical significance was determined by one-way ANOVA; ***p < 0.001 and n.s., not significant.

During SARS-CoV-2 infection, spike-ACE2 binding facilitates ACE2 cleavage by TMPRSS2 and ADAM17, resulting in the production of the ACE2 ectodomain^45,46^. On the other hand, this interaction triggers the internalization of ACE2 for SARS-CoV-2 entry^2^. Because incoming viral particle-derived NPs accumulate on the plasma and endosomal membranes in the presence of UNI418 (Fig. 5d, middle panels), we hypothesized that the compound could affect ACE2 cleavage or internalization. To test this hypothesis, we analyzed ACE2 levels in SARS-CoV-2-infected cells after UNI418 treatment by Western blot analysis. Consistent with this hypothesis, full-length ACE2 (approximately 110 kDa) was reduced by SARS-CoV-2 infection at 1 and 3 h postinfection, accompanied by the appearance of an ACE2 ectodomain of approximately 75 kDa (Fig. 6a, compare Lanes 1 and 2, or Lanes 7 and 8). Interestingly, UNI418 treatment inhibited the decrease in endogenous full-length ACE2 caused by SARS-CoV-2 infection in a dose-dependent manner (Fig. 6a, Lanes 3 to 6 and Lanes 9 to 12). To verify the role of UNI418 in ACE2-mediated endocytosis, we incubated hACE2-GFP-expressing HEK293T cells with spike-RBD for 1 or 2 h and monitored the internalization of hACE2-GFP. After 2 h of incubation with spike-RBD, hACE2-GFP was mostly detected in cytosolic vesicles, indicating ACE2-mediated endocytosis (Fig. 6b, top and second rows). UNC3230, UNI418 and dynasore (a known inhibitor of endocytosis) successfully inhibited the ACE2-mediated endocytosis triggered by spike-RBD (Fig. 6b, third, fifth and bottom rows), but apilimod did not obviously inhibit this process (Fig. 6b, fourth row). These results were quantified by the ratio between the internalized and membranous hACE2-GFP signals in individual cells, and the results showed the same conclusion (Fig. 6c). These results suggested that UNI418 inhibited ACE2-mediated endocytosis of spike-RBD-containing SARS-CoV-2 through PIP5K1C rather than through PIKfyve.

### UNI418 inhibits SARS-CoV-2 spike-mediated pseudovirus entry through PIP5K1C and PIKfyve

**Fig. 7:**
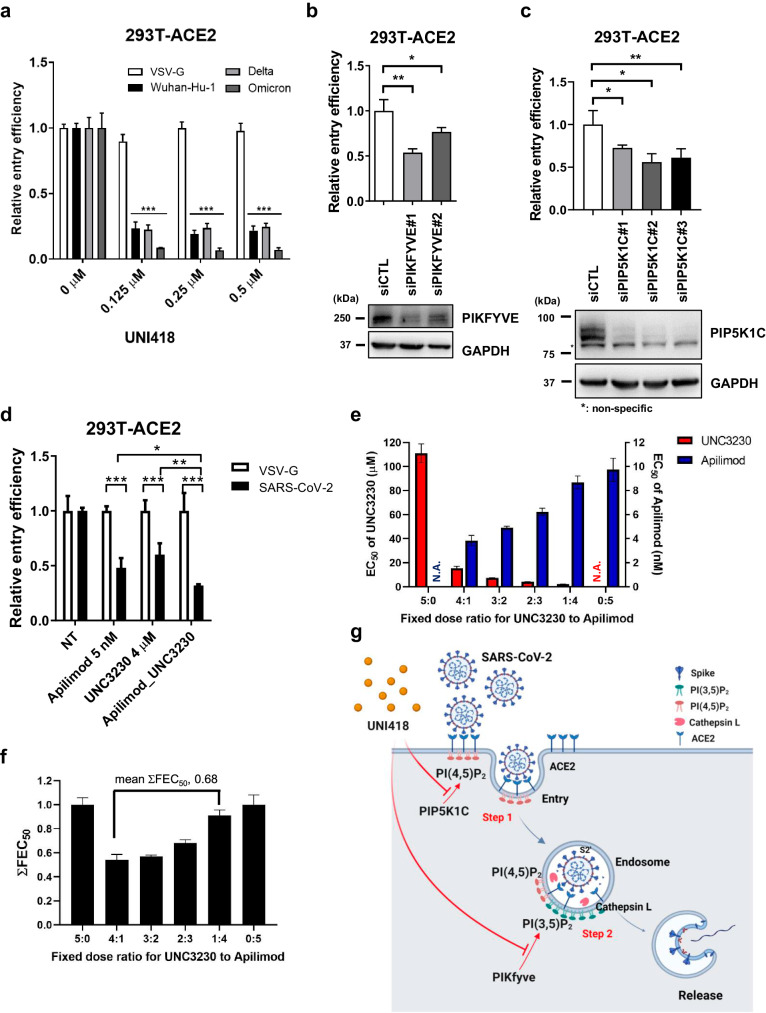
PIP5K1C and PIKfyve are key regulators of SARS-CoV-2 spike protein-mediated viral entry. **a** Pseudotyped virus entry assay in the presence of the indicated concentrations of UNI418. Expression plasmids for VSV-G and the SARS-CoV-2 spike protein from WuHan-Hu-1, the Delta variant (B.1.617.2) and the Omicron variant (BA.1) were used to produce pseudotyped virus particles with a luciferase reporter. The 293T-ACE2 cells were infected with each pseudotyped virus under the indicated concentrations of UNI418. The relative viral entry efficiency was determined by the luciferase activity 24 h postinfection and is presented as the mean ± SEM, *n* = 3. Statistical significance was determined by two-way ANOVA, with ***p < 0.001. **b**, **c** Entry of Wuhan-Hu-1 spike-pseudotyped virus was assessed after PIKfyve and PIP5K1C knockdown in 293T-ACE2 cells. HEK293T-ACE2 cells were transfected with the indicated siRNAs and subjected to a luciferase-based virus entry assay (top panel) and Western blot analysis (bottom panel). The data are presented as the means ± SEMs, *n* = 3. Statistical significance was determined by Student’s *t*-test, with *p < 0.05 and **p < 0.01. **d** Entry of Wuhan-Hu-1 spike-pseudotyped virus was measured by luciferase activity after treatment with apilimod or UNC3230 and their cotreatment in 293T-ACE2 cells. The data are presented as the means ± S.D.s, *n* = 3. Statistical significance was determined by Student’s t test, with *p < 0.05, **p < 0.01 and ***p < 0.001. **e**, **f** Synergistic effect of UNC3230 and apilimod against SARS-CoV-2 infection. Vero cells infected with SARS-CoV-2 at an MOI of 0.01 were treated with 3-fold serial dilutions of different ratios of UNC3230 and apilimod: 5:0 (300 µM UNC3230 only), 4:1 (240 µM UNC3230 and 60 nM apilimod), 3:2 (180 µM UNC3230 and 120 nM apilimod), 2:3 (120 µM UNC3230 and 180 nM apilimod), 1:4 (60 µM UNC3230 and 240 nM apilimod), and 0:5 (300 nM apilimod only). **e** On Day 1 after infection, the antiviral efficacy of UNC3230 (red) and apilimod (blue) was individually determined in each combination. **f** Isobologram graph showing the sums of fractional EC_50_ values (ΣFEC_50_) for each combination. The mean value from the fixed dose ratios of 4:1, 3:2, 2:3 and 1:4 was 0.68. **g** The graphical model shows how UNI418 inhibits SARS-CoV-2 entry into cells by targeting PIP5K1C and PIKfyve.

To further clarify the inhibitory role of UNI418 in SARS-CoV-2 entry, we used a SARS-CoV-2 spike-pseudotyped lentivirus system featuring a luciferase reporter^2^. Specifically, replication-deficient lentiviruses harboring the SARS-CoV-2 spike protein (referred to as SARS-CoV-2 pseudoviruses) were used to assess their entry into hACE2-expressing HEK293T cells. This pseudovirus assay enabled us to focus on the viral entry step that is mediated by SARS-CoV-2 spike and hACE2 interactions while excluding other steps of the viral infection cycle, such as genome replication or assembly. To ensure reliability and broad-spectrum antiviral activity, we performed a pseudovirus entry assay using different spike proteins derived from the original Wuhan-Hu-1, Delta variant (B.1.617.2), and Omicron variant (BA.1) strains, with VSV-G used as the control. UNI418 treatment markedly inhibited the entry of Wuhan-Hu-1, Delta, and Omicron variant pseudotyped viruses (Fig. 7a). In contrast, VSV-G-pseudotyped viral entry was unaffected by UNI418 treatment.

Since UNI418 specifically inhibits PIP5K1A, PIP5K1B, PIP5K1C, and PIKfyve (Fig. 1b), we examined whether genetic knockdown of each gene inhibited SARS-CoV-2 spike-mediated viral entry. Knockdown of PIP5K1C and PIKfyve significantly inhibited SARS-CoV-2 (Fig. 7b, c), while knockdown of either PIP5K1A or PIP5K1B did not (Supplementary Fig. 14a, b). These results strongly suggested that PIP5K1C and PIKfyve, but not PIP5K1A/B, were proviral factors that assisted in SARS-CoV-2 cellular entry.

To examine the synergistic effect of dual inhibition of PIKfyve and PIP5K1C, we performed a pseudovirus entry assay with apilimod and/or UNC3230. First, we identified a compound concentration that inhibited SARS-CoV-2 spike-mediated viral entry by 50% compared to maximal inhibition. These concentrations were 5 nM for apilimod and 4 μM for UNC3230 (Supplementary Fig. 14c, d). As shown in Fig. 7d, dual treatment with apilimod and UNC3230 more efficiently inhibited SARS-CoV-2 spike-mediated viral entry than single treatments, indicating the synergistic effect of dual inhibition of PIKfyve and PIP5K1C. This finding was reproducible in SARS-CoV-2-infected cells after treatment with UNC3230 and apilimod in different combinations on Day 1 (Fig. 7e). The results showed that the mean of the sums of the fractional EC_50_ indices (ΣFEC_50_) was 0.68, indicating that the two compounds were synergistic, as ΣFEC_50_ values below 0.8 are considered synergistic (Fig. 7f).

Taken together, these findings indicated that UNI418 reduced the level of PtdIns(4,5)P2 in the plasma membrane by inhibiting PIP5K1C, resulting in the blockade of ACE2-mediated endocytosis of SARS-CoV-2 (Fig. 7g, Step 1). In addition, UNI418 inhibited PIKfyve-mediated PtdIns(3,5)P2 synthesis, which is known to be important for the proteolytic maturation of cathepsin L. This inhibition prevented endosomal escape of the SARS-CoV-2 genome, which was facilitated by cathepsin L-mediated maturation of the spike protein into a fusogenic protein (Fig. 7g, Step 2).

## Discussion

Since the emergence of SARS-CoV-2, which caused the coronavirus disease outbreak in 2019, several neutralizing antibodies, vaccines, and therapeutic drugs have been developed or are currently in clinical studies to protect against SARS-CoV-2 as well as its variants. Although certain therapeutic strategies that directly target viral proteins are effective, they are often accompanied by the risk of failure because of the rapid mutation of viral RNA genomes and the potential emergence of drug-resistant strains. Neutralizing antibodies and vaccines developed during the early phase of the outbreak have become less potent or have diminished efficacy in preventing the widespread transmission of mutant strains, including the Omicron variant^47^. Thus, it is important to develop additional small molecule-based antiviral agents with different chemical skeletons or modes of action against SARS-CoV-2 infection. In this study, we propose UNI418, which targets both PIP5K1C and PIKfyve, as a novel therapeutic compound against SARS-CoV-2 infection (Fig. 5).

Although the PIPK profiling assay revealed PIP5K1B, PIP5K1C, and PIKfyve as the main targets of UNI418 (Fig. 1b), genetic ablation of *PIP5K1B* did not inhibit viral entry into the SARS-CoV-2 spike-pseudotyped virus (Supplementary Fig. 14a). In contrast, PIP5K1C or PIKfyve knockdown successfully inhibited entry, indicating that these two PIPKs could represent functional targets for the antiviral effects of UNI418 (Fig. 7b, c). It has been reported that inhibition of PIKfyve (by apilimod) prevents viral cytoplasmic entry and infection by SARS-CoV-2^20^. More recently, it has been suggested that the *PIP5K1C* mRNA transcript level is increased after SARS-CoV-2 infection of tonsil epithelial organoids established from human tissues^48^. This preliminary transcriptome analysis suggested that PIP5K1C may primarily participate in the remodeling of cellular lipid metabolism upon viral infection. Here, we identified PIP5K1C as a potential target for inhibiting SARS-CoV-2 infection. Given the accumulating evidence supporting the validation of PIP5K1C as a viable target, evaluating the antiviral efficacy of UNI418 using an ex vivo human organoid challenge model as a preclinical approach would be worthwhile.

PIKfyve mediates the synthesis of lysosomal PtdIns(3,5)P_2_ and PtdIns(5)P, which are important for late endosomal and lysosomal functions and homeostasis^33^. PIKfyve-mediated synthesis and turnover of lysosomal PtdIns(3,5)P_2_ are key regulatory molecular mechanisms in autophagosome–lysosome fusion^33^. We showed that PIKfyve inhibition by UNI418 and apilimod led to the inhibition of autophagosome-lysosome fusion (Figs. 2 and 3a–c). In addition, lysosomal targeting and subsequent proteolytic maturation of cathepsins were inhibited by UNI418 and apilimod (Fig. 4a–c), indicating that PIKfyve was responsible for this effect. The membrane fusion between SARS-CoV-2 and endosomes/lysosomes, as well as the subsequent endosomal release of the SARS-CoV-2 genome, absolutely rely on proteolysis of the spike protein into a mature fusogenic protein by host cathepsin L within late endosomes/lysosomes^12–14^. We demonstrated that UNI418 inhibited the maturation of cathepsin L (Fig. 4d, e), resulting in a delay in proteolytic activation of the spike protein (Fig. 4f, g). However, several reports have raised concerns that inhibiting PIKfyve with its antagonists, such as apilimod, blocks the transport of MHC class II to the cell surface, thus reducing antigen presentation^49^. This inhibition also negatively affects Toll-like receptor (TLR)-mediated production of antiviral type I interferons by modulating activating transcription factor 3 (ATF3)^50^. Furthermore, it exacerbates the already impaired T-cell immunity observed in SARS-CoV-2-infected patients, resulting in decreased production of proinflammatory cytokines that are crucial for the host defense mechanism^51,52^. Therefore, dose optimization of PIKfyve inhibitors or their combination with an immunostimulatory molecule might be necessary, particularly when they are used as antiviral agents.

PIP5Ks, including the isozymes PIP5K1A, PIP5K1B, and PIP5K1C, are key enzymes involved in the conversion of PtdIns4P to PtdIns(4,5)P_2_. This lipid is required for clathirin-mediated endocytosis^32,33^, which is a common route of viral entry^16,17^. Thus, we suggested that UNI418 reduced the level of PtdIns(4,5)P_2_ by locally inhibiting PIP5K1C (Supplementary Fig. 3), resulting in the blockade of ACE2-mediated endocytosis of SARS-CoV-2 (Fig. 6). In addition, we observed that UNI418 and UNC3230 deacidified endolysosomes (Fig. 3d, e), which warrants further investigation to determine their relationship with endosomal escape of the viral genome. It also remains to be elucidated why the knockdown of only PIP5K1C and not PIP5K1A/B inhibited SARS-CoV-2 spike-pseudotyped viral entry. Based on these findings, we suggest that UNI418 has pleiotropic effects on SARS-CoV-2 infection through dual targeting of PIKfyve (which inhibits endosomal escape of SARS-CoV-2) and PIP5K1C (which inhibits ACE2-mediated endocytosis of SARS-CoV-2).

In summary, we demonstrated that UNI418, a novel PIKfyve and PIP5K1C dual-target inhibitor, successfully suppressed SARS-CoV-2 infection at nontoxic concentrations. Mechanistically, UNI418 inhibited ACE2-mediated endocytosis and cathepsin L-mediated endosomal escape of SARS-CoV-2. Our findings suggest that pharmacological inhibition of PIKfyve and the novel PIP5K1C could represent an effective therapeutic strategy for preventing infection by SARS-CoV-2 or its variants and for preparing for future pandemics.

## Supplementary information

Supplementary Information
