# Supplementary material for: A dual inhibitor of PIP5K1C and PIKfyve prevents SARS-CoV-2 entry into cells
Source: Exp Mol Med. 2024 Aug 1;56(8):1736–49. doi: 10.1038/s12276-024-01283-2 (PMC11372076; doi:10.1038/s12276-024-01283-2)
Supplement: Supplementary file 1 — Supplementary Information [file 12276_2024_1283_MOESM1_ESM.pdf]

A dual inhibitor of PIP5K1C and PIKfyve prevents SARS-CoV-2 entry into cells

Supplementary Information

Yuri Seo[1,2†], Yejin Jang[3†], Seon-gyeong Lee[2,5,8†], Joon Ho Rhlee[4], Sukyeong Kong[2,6], Thi Tuyet Hanh Vo[1], Myung hun Kim[1], Myoung Kyu Lee[3], Byungil Kim[3], Sung You Hong[2,4], Meehyein Kim[3,7*], Joo-Yong Lee[1*], and Kyungjae Myung[2,6*]

*Corresponding author: Kyungjae Myung, E-mail: kmyung@ibs.re.kr; Joo-Yong Lee, E-mail: leejooyong@cnu.ac.kr; Meehyein Kim, E-mail: mkim@krict.re.kr

Supplementary Materials and Methods

Western blot

The whole cell extracts were prepared by lysis of harvested cells with M-PER Mammalian protein extraction reagent (Thermo Fisher Scientific). The protein concentrations were determined by Bradford protein assay (Bio-Rad). The 10 to 20 μg of prepared proteins were separated by the SDS-PAGE and transferred to PVDF membrane, which were then blocked in 5% skim milk for 30 minutes at room temperature. Membranes were incubated with a primary antibody detecting interest protein at 4°C overnight. After washing with Phosphate-Buffered Saline (PBS) with 0.1% Tween (PBS-T), the membranes were incubated with a secondary antibody for 1 h at room temperature. The images were obtained and quantified by Fusion solo ChemiDoc system (Vilber).

Autophagic flux assay

Autophagic flux assay was analyzed essentially as previously described[1,2]. Briefly, RPE1 cells were treated with UNI418 (0, 0.5, or 1 μM) for 2 h with/without bafilomycin A_1 to inhibit lysosomal degradation under normal growth media or HBSS. The treated cells were subjected to western blot analysis using LC3 and GAPDH antibodies. The LC3-II bands were quantified using Fusion Solo ChemiDoc system (Vilber) and normalized to GAPDH bands. Autophagy flux was calculated by subsidizing the lysosomal inhibitor-treated cells to non-treatment cells in both normal growth media and HBSS-induced starvation with 0, 0.5, or 1 μM UNI418.

Antibodies

The following antibodies were used for western blotting and immunofluorescence; Rabbit polyclonal LC3B (Cell Signaling Technology, 2775S), LC3B (Sigma-Aldrich, L7543), SQSTM1/p62 (Santa Cruz

Biotechnology, sc-25575), PIP5K1C (Cell Signaling Technology, #3296), EEA1 (Santa Cruz Biotechnology, sc-33585), LAMP1 (Cell Signaling Technology, #9091) and Mouse monoclonal SARS-CoV/SARS-CoV-2 (COVID-19) Spike (GenTex, GTX632604), SARS-CoV/SARS-CoV-2 nucleocapsid (Sinobiological, 40143-MM05), PIP5K1B (Santa Cruz Biotechnology, sc-514169), PIKfyve (EMD Millipore, MABS522), ACE2 (R&D systems, MAB933), GAPDH (Santa Cruz Biotechnology, sc-32233), cathepsin D (Santa Cruz Biotechnology, sc-377124), cathepsin L (eBioscience, BMS1032), EEA1 (Cell Signaling Technology, # 48353), PIP2 (Santa Cruz Biotechnology, sc-53412).

Plasmid Construction

Plasmids encoding the SARS-CoV2 HexaPro were provided by Ho Min Kim (Institute for Basic Science). The Spike protein from the SASR-CoV-2 (pcDNA3.1-SARS2-Spike) was a gift from Fang Li (Addgene plasmid # 145032). pBOBI-FLuc, pcDNA3.3-SARS2-B1.617.2 and pcDNA3.3_SARS2_omicron_BA.1 were a gift from David Nemazee (Addgene plasmid # 170674, Addgene plasmid # 172320 and Addgene plasmid # 180375). GFP-PIPK1 gamma 90 was a gift from Pietro De Camilli (Addgene plasmid # 22299) and GFP-hPIKfyve was a gift from Geert van den Bogaart (Addgene plasmid # 121148). pcDNA3.1-ACE2-GFP was a gift from Utpal Pajvani (Addgene plasmid # 154962). mCherry-Rab5 and-Rab7A were a gift from Gia Voeltz (Addgene plasmids # 49201 and # 61804, respectively). mCherry-Lysosomes-20 was a gift from Michael Davidson (Addgene plasmid # 55073). pMD2.G was a gift from Didier Trono (Addgene plasmid # 12259).

siRNA

The sequence of siRNA oligonucleotides are as follows: Control siRNA 5’-UUCAAUAAAUUCUUGAGGUUU-3’, PIP5K1B#1 siRNA 5’-CAGCAAAGGGUUACCUUCCAGUUCA-3’, PIP5K1B#2 siRNA 5’-

CCAUUAGCAUUCCGAUAUU-3’, PIP5K1B#3 siRNA 5’-GCCACCTTCTTTCGAAGAA-3’, PIP5K1C#1 siRNA 5’-GCGUGGUCAAGAU GCACCUCAAGUU-3’, PIP5K1C#2 siRNA 5′- GCUACUACAU GAACCUCAACCAGAA -3′, PIP5K1C#3 siRNA 5’-CAUUUAAGGACUUAGAUUU -3, PIKfyve#1 siRNA 5'- AACCUUUGACUC CUGAUCAAGAUGA -3 and PIKfyve#2 siRNA 5'-CCACAGACAGUAAUUCUAUUGGGGA -3'. siRNA oligonucleotides were purchased from Genolution, Sigma-Aldrich and Integrated DNA Technologies. These siRNAs were transfected into the cell using lipofectamine RNAiMAX (Invitrogen, 13778150) following manufacturer’s protocol.

Cell imaging

RPE1 cells were treated with UNI418 at indicated concentration for over-night. After that, the image was taken by EVOS M5000 (Thermo Fisher Scientific).

Vacuolization analysis

RPE1 cells were treated with UNI418 at the different concentration during the time. Live cell images were taken by Lionheart LX Automated Microscope and analyzed in Gen5 (Agilent).

Flow cytometry

RPE1 cells were treated with indicated doses of UNI418 overnight. To analyze PIP2 level by flow cytometry, trypsinized cells were fixed with 4% paraformaldehyde for 10 min at room temperature. The fixed cells were permeabilized with 0.2% PBS-T (Triton X-100) for 5min and blocked with 5% Bovine Serum Albumin (BSA), and stained with the primary antibody for 4h at 4°C. Cells were washed three times with 1% BSA-PBS and incubated with the fluorescence-conjugated secondary antibody Alexa Fluor® 488 AffiniPure Goat Anti-Mouse IgG (H+L) (Jackson ImmunoResearch,111-545-003) for 1h at room temperature analyzed by BD Accuri C6 Plus flow cytometer (BD Biosciences).

Quantitative RT-PCR

Vero cells seeded on 6-well plates (at a density of 5 × 10^5 cells per well) were incubated overnight and infected with SARS-CoV-2 (MOI, 0.001) for 1 h. After washing with PBS twice, they were treated with DMSO as a mock or increasing concentrations of a compound, either UNI418 or Apilimod. At 24 h, culture supernatants were harvested for viral RNA purification using the QIAmp Viral RNA Mini kit (Qiagen, 52904). Viral RNA was quantified using a one-step real-time RT-PCR kit with an *NP*-gene specific primer set (PCL Inc.). For checking PIP5K1A knockdown, total RNA was isolated using AccuPrep Universal RNA extraction Kit (Bioneer, K-3140) following the manufacturer's protocol. cDNA was synthesized by ReverTra Ace qPCR RT Master Mix with gDNA remover (Toyobo, FSQ-301) following the manufaturer's protocol. Relative mRNA quantification was measured by SYBR green (Toyobo, QPK-201) using CFX96 Real-Time System (Biorad). β-actin was used as an international control. Primer used for PCR; PIP5K1A (Forward 5'-ACTTACCAGCCATCGGTCTCTG-3' and Reverse 5'-ACATCAGGACGACCAAGGTGAAC-3'), PIP5K1C (Forward 5'-GCTTCCCCACCTACAAGGAC-3' and Reverse 5'-GTGGAGTAGAGCGCCTTCTG-3') and PIKFYVE (Forward 5'-ACCATTGCCGACTATGTGGG-3' and Reverse 5'-AGGTGTTCGGGGTTCACTTG-3').

Immunofluorescence

Cells were plated on 22mm × 22mm cover glass in 6 well plate and incubated for one day. The cells were treated with indicated chemicals over-night. To visualize chromatin bound proteins by immunofluorescence, PBS washed cells were fixed with 4% paraformaldehyde for 15 min at room temperature. The fixed cells were permeabilized with PBS with 0.2% Triton X-100 (PBS-T) for 5 min at room temperature and blocked with 5% Normal Goat Serum (NGS [Gibco,

PCN5000]), and stained with the primary antibody for overnight at 4°C. Cells were washed three times with 0.1% PBS-T and incubated with the fluorescence-conjugated secondary antibodies Rhodamine Red™-X (RRX) AffiniPure Goat Anti-Mouse IgG (H+L) (Jackson ImmunoResearch,115-295-062), Alexa Fluor® 488 AffiniPure Goat Anti-Rabbit IgG (H+L) (Jackson ImmunoResearch,111-545-003) for 1h at room temperature followed by mounting in permanent mounting medium with DAPI (Southernbiotech, 0100-20). For LAMP1 staining, cells were fixed and permeabilized with ice-cold methanol for 10 min, and blocked with 10% goat serum in PBS for 30 min at room temperature. Then cells were stained with LAMP1 antibody in PBS with 10% goat serum for overnight at 4℃. Cells were washed three times with 10% goat serum in PBS and incubated with secondary antibodies for 1hr at room temperature followed by mounting in permanent mounting medium with DAPI. The images were taken with BX53 (Olympus) or LSM 880 confocal microscope (Carl Zeiss). For quantification of colocalization index (Pearson's correlation coefficient), images were analyzed by ImageJ softwere (MD) at least 45 cells per each group.

Statistical analysis

The statistical analysis for each experiment is described in its corresponding figure legends. Generally, statistical analysis was performed by One-way ANOVA or Two-way ANOVA or *Student's t-test* using GraphPad Prism software (GraphPad Software). Data are presented as means ± SEM of duplicate or triplicate samples and significance was set at *$p < 0.05$, **$p < 0.01$, ***$p < 0.001$ and ****$p < 0.0001$.

Supplementary Figures

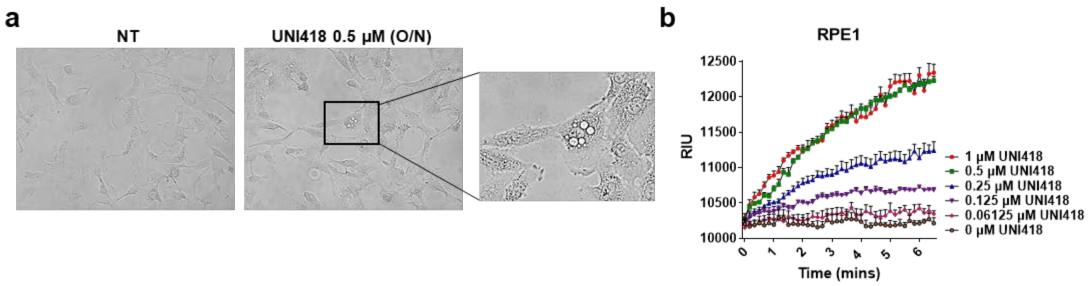

Supplementary Fig. 1 UNI418 induces the vacuolization in RPE1 cells. a Cell image of RPE1 cells with/without UNI418 treatment. **b** Graph of vacuolization analysis in RPE1 cells at the different dose. Data presented as mean ± SEMs, n=4.

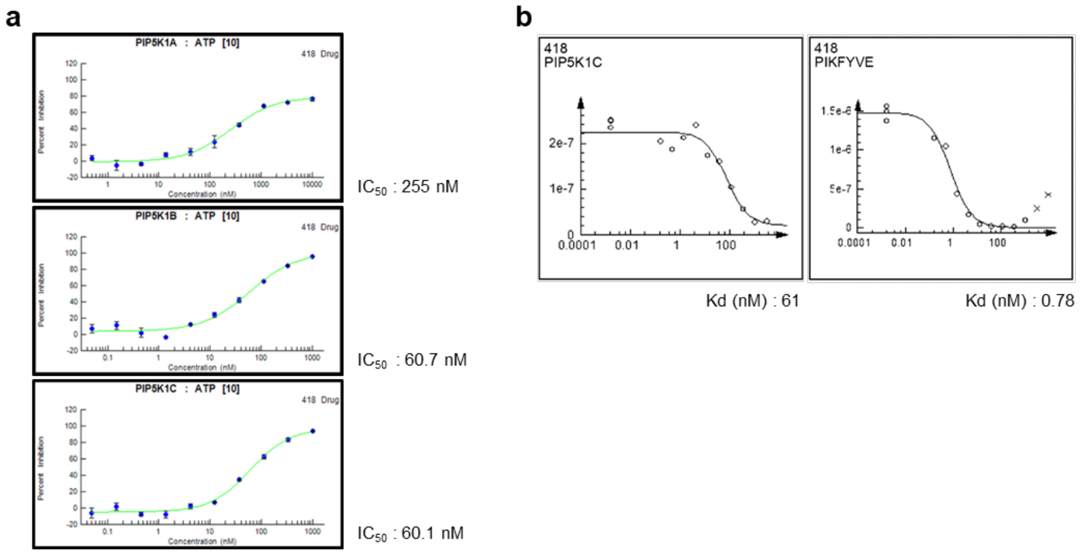

Supplementary Fig. 2 UNI418 inhibits PIP5K1B/C and PIKfyve. a Inhibition graph of PIP5K1 kinases at the 10 point titration with UNI418. IC_{50} value of PIP5K1 kinases was analyzed by Thermo Fisher Scientific's SelectScreen kinase profiling service. **b** K_d (nM) values of PIP5K1C and PIKfyve inhibition were determined using the Eurofins kinase assay service.

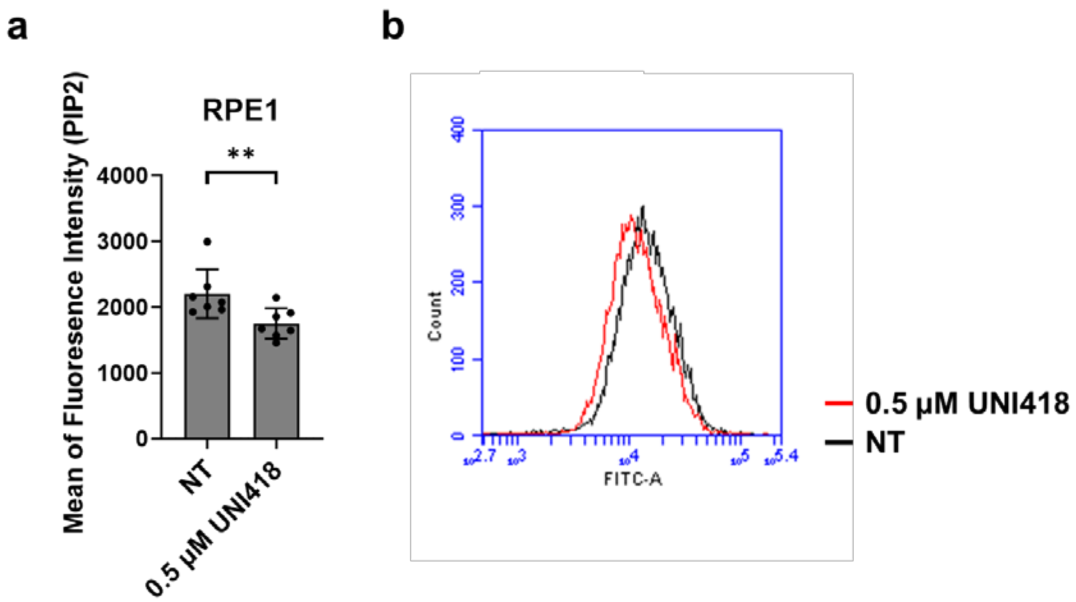

Supplementary Fig. 3 UNI418 reduces the amount of PIP2 in RPE1 cells. **a** PtdIns(4,5)P_2 level in RPE1 cells with/without UNI418 treatment. PtdIns(4,5)P_2 level was analyzed by flow cytometry. Data presented as mean ± SEMs, n=7. Significance was determined by Student's t-test, $^{**}p < 0.01$. **b** Representative flow cytometry graph for PIP2 level after indicated dose of UNI418 treatment.

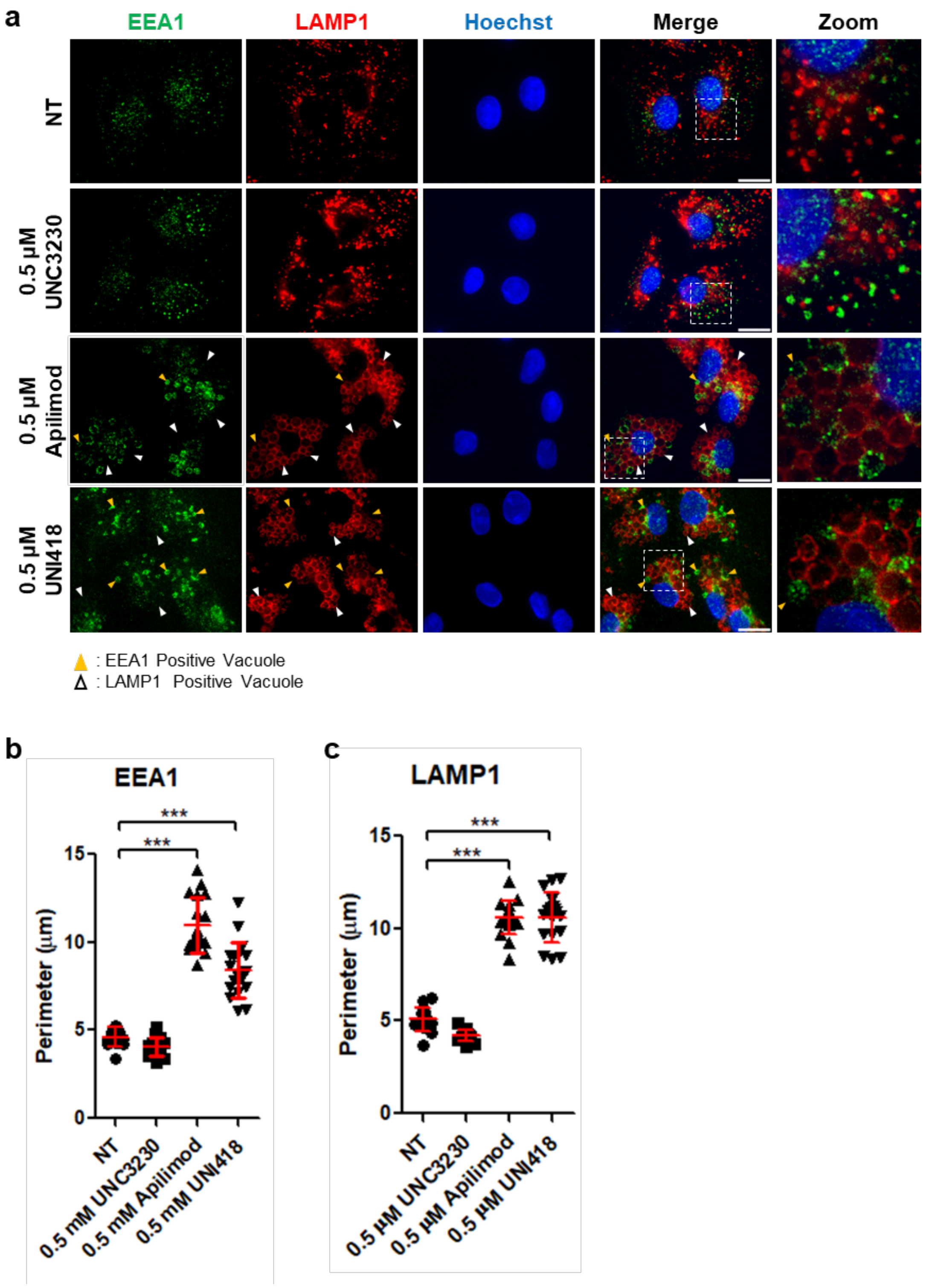

Supplementary Fig. 4 PIKfyve is responsible for the vacuolization induced by UNI418 treatment. a Immunofluorescence staining showing EEA1 (green, yellow arrowhead) and LAMP1 (red, white arrowhead) in RPE1 cells with UNC3230, Apilimod and UNI418 treatment, and images within the white rectangular boxes are zoomed in on the right side. Scale bar, 15 µm. **b, c** Quantification of EEA1(**b**) and LAMP1 (**c**) positive vacuole perimeter under

indicated drug treatment. Data were presented as mean ± SEMs, n >20. Significance was determined by Student's t-test, ***$p < 0.001$.

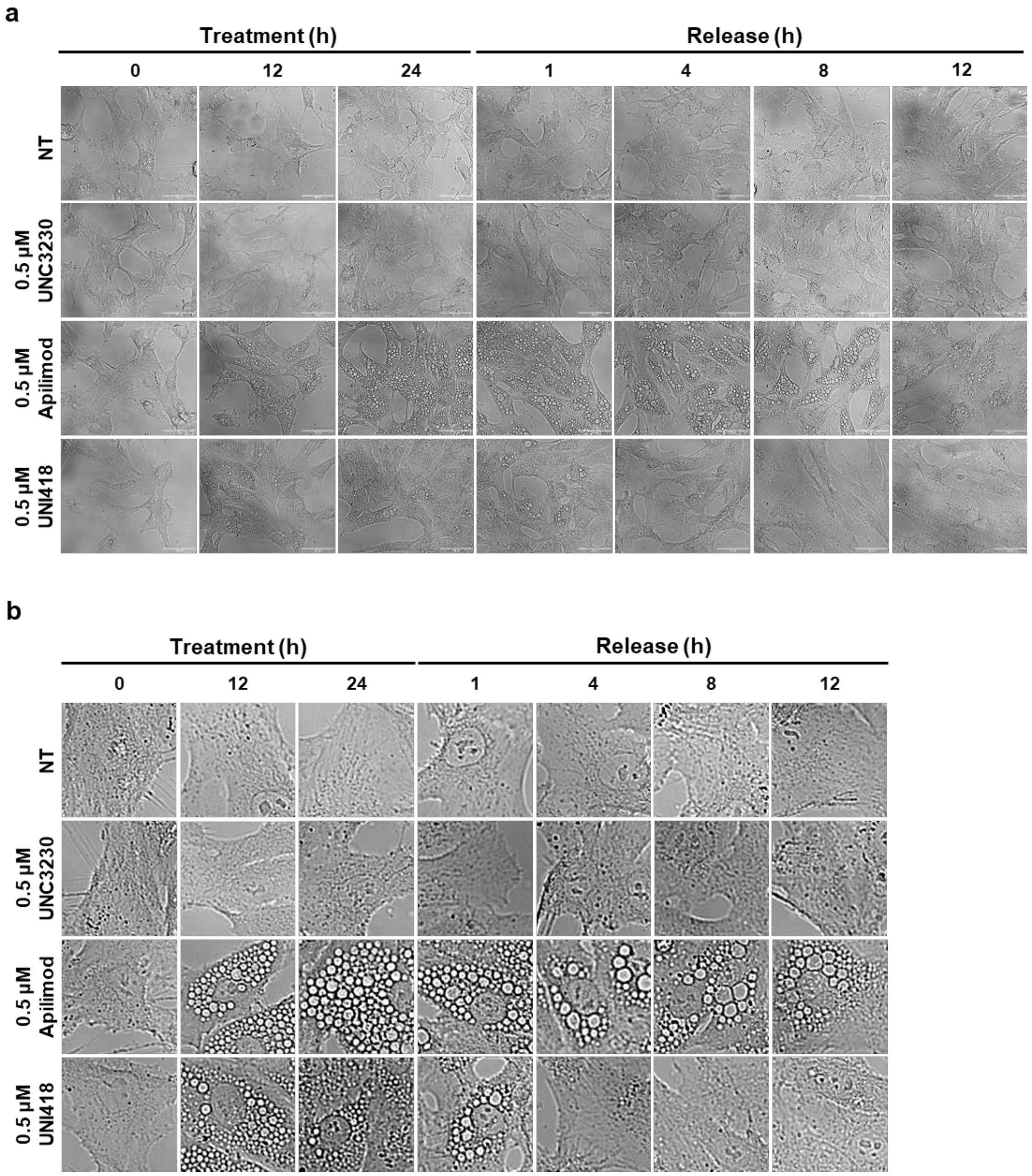

Supplementary Fig. 5 The vacuolization triggered by PIKfyve inhibition is reversible. a Representative image at the each time point after UNC3230, Apilimod and UNI418 treatment or release in RPE1 cells. Scale bar, 50 µm. **b** Enlarged image (Zoom) of specific area in the upper images.

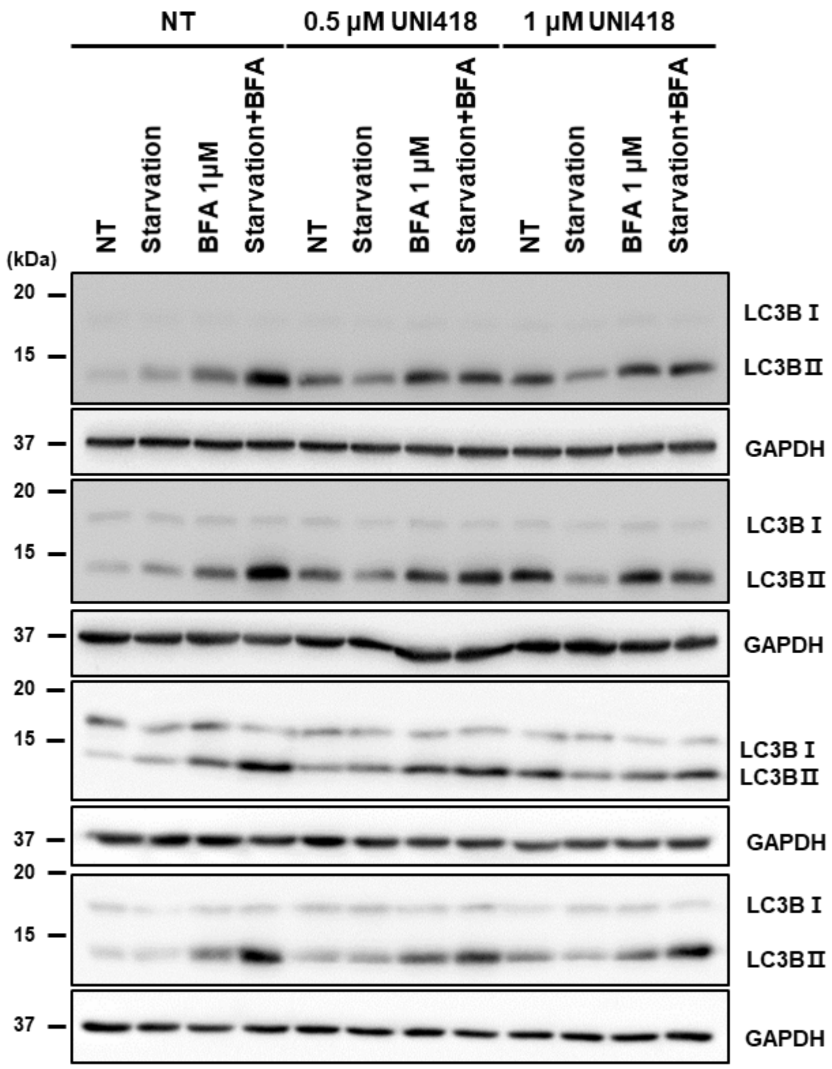

Supplementary Fig. 6. Western blot results for the autophagy flux assay in Fig 2e.

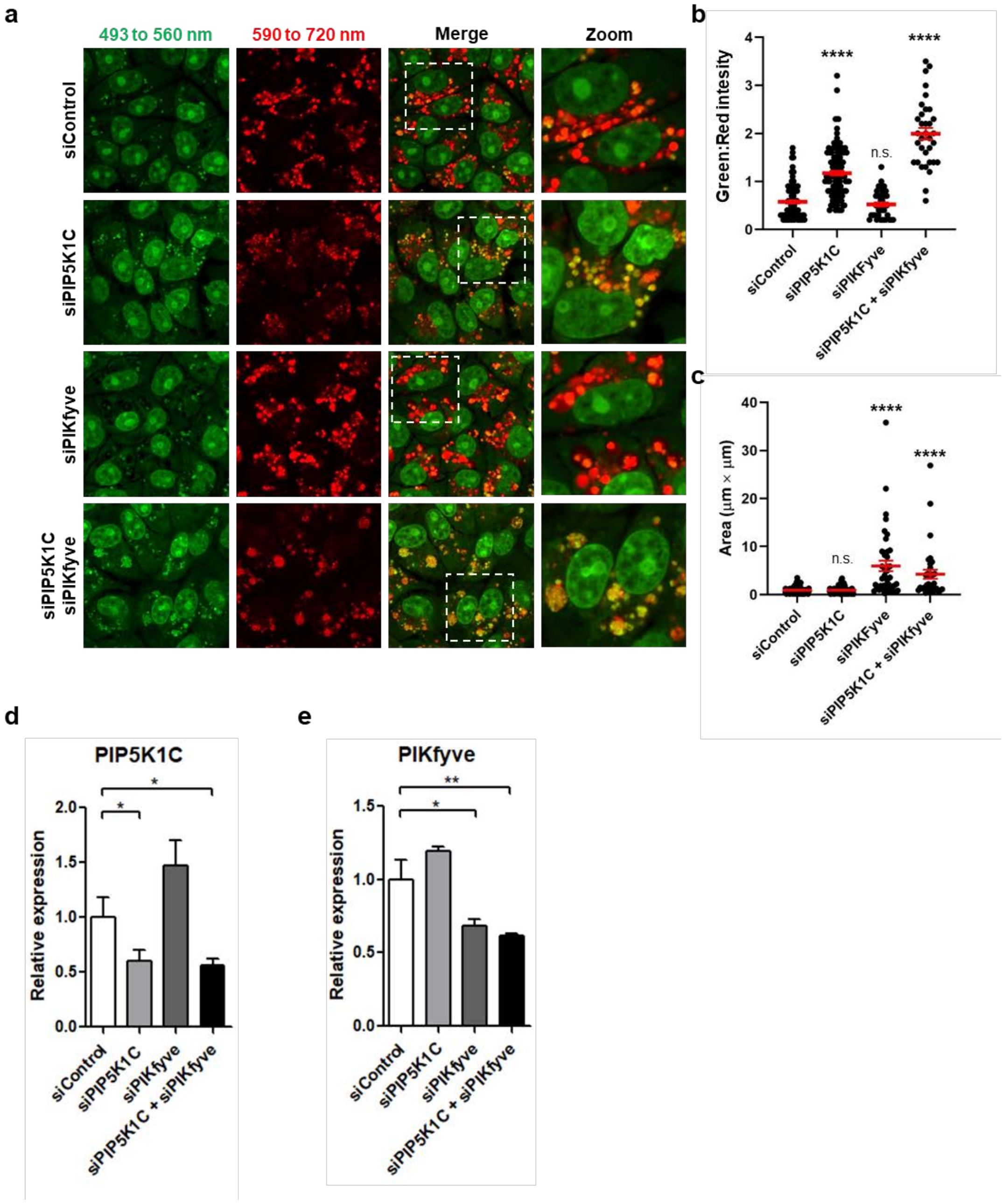

Supplementary Fig. 7. Inhibition of endo-lysosomal acidification by PIP5K1C siRNA and vacuole enlargement by PIKfyve siRNA. a Vero cells were treated with control siRNA (siControl), PIP5K1C siRNA (siPIP5K1C), PIKfyve siRNA (siPIKfyve) or the combination of siPIP5K1C and siPIKfyve at a concentration of 50 nM. At day 2 after transfection, acridine orange (4 µg/mL) was added to the transfected cells for 30 min. Fluorescent images were captured by confocal microscopy with two emission wavelengths at 493-560 (green) and 590-720 nm (red) after excitation at 488 nm. Merged images are presented, and their regions of interest (ROIs) marked in white boxes are

zoomed in on the right. Original magnification, ×630. Green-to-red fluorescent intensity ratio (**b**) and area of the fluorescent spots (**c**) were quantified. Data presented as mean ± SEMs, n >30. **d**, **e** The graph for checking the expression of PIP5K1C (**d**) and PIKfyve (**e**) using real-time PCR. Data presented as mean ± SEMs, n=3. (**b**, **c**, **d** and **e**) Significance was determined by Student's t-test, *$p < 0.05$, **$p < 0.01$, ****$p < 0.0001$ and n.s., not significant.

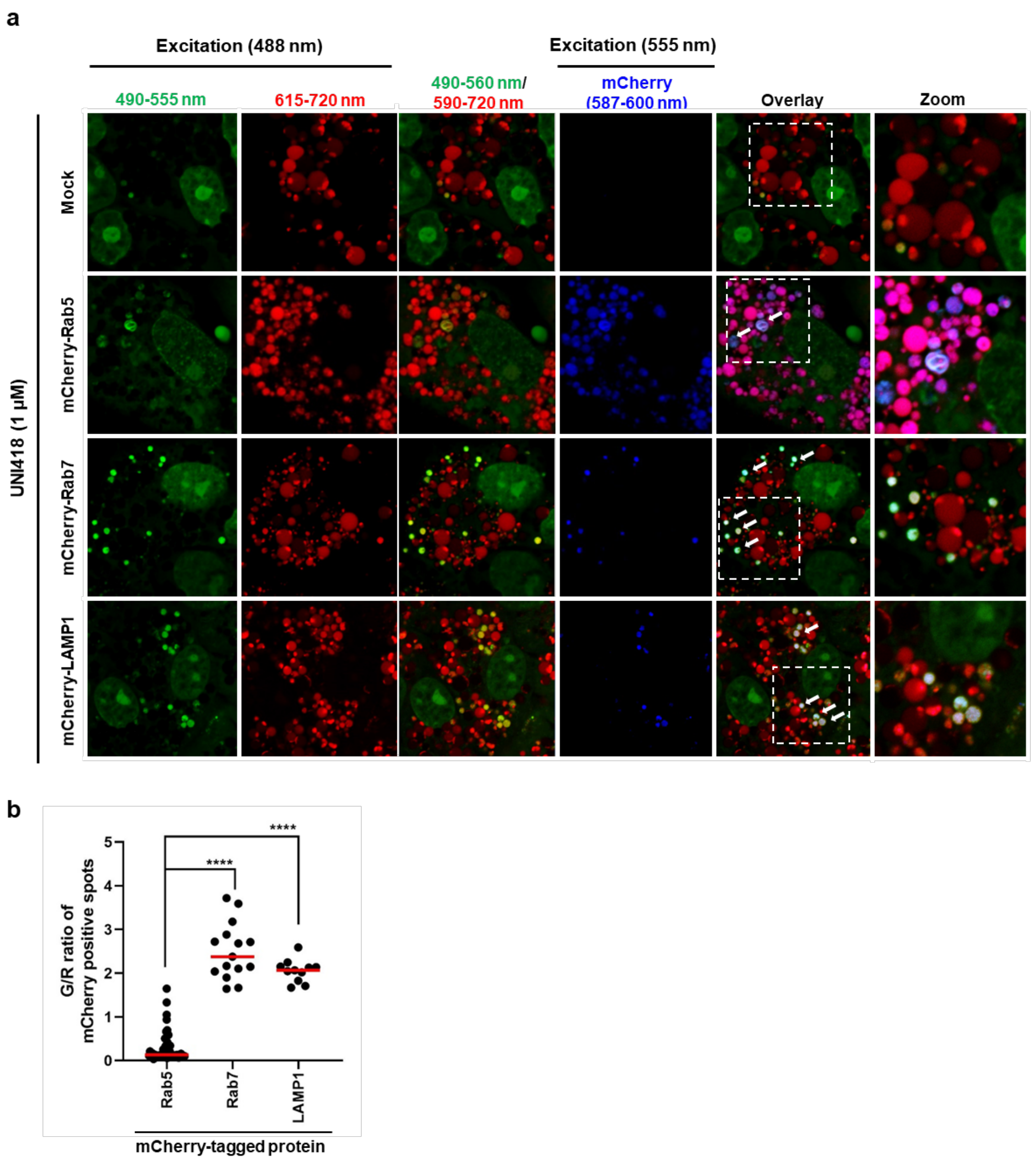

Supplementary Fig. 8 Identification of deacidified vesicles by UNI418. a Vero cells were mock-transfected (Mock) or transfected with plasmids expressing mCherry-fused Rab5 (mCherry-Rab5), Rab7 (mCherry-Rab7) and LAMP1 (mCherry-LAMP1). On day 2 after transfection, they were treated with 1 µM UNI418 for 1 h and then stained with acridine orange (AO) for 30 min. AO-derived fluorescence was detected using two band pass filters, 490-555 nm (green) and 615-720 nm (red), after excitation at 488 nm. Using the same samples, markers for early endosomes (Rab5), late endosomes (Rab7) and lysosomes (LAMP1) were visualized at 587-600 nm after excitation at 555 nm. Deacidified vesicles are pointed out with white arrows on the overlay images.

The regions of interest (ROIs) marked in white boxes are displayed in a zoomed-in view on the right. **b** The graph displaying green-to-red (G/R) ratios of mCherry positive spots. Statistical significance was determined using an unpaired t-test, ****$p < 0.0001$.

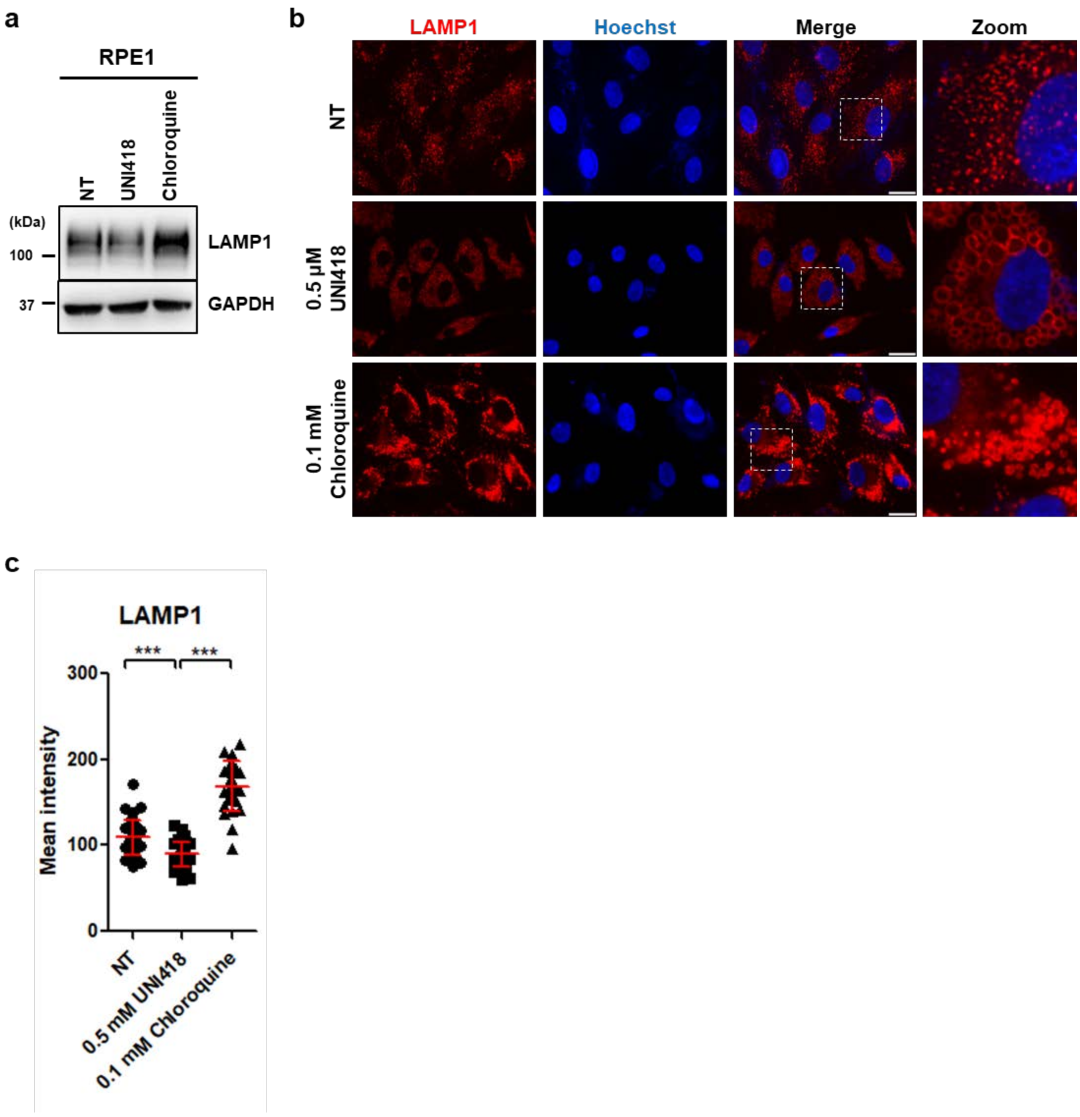

Supplementary Fig. 9 UNI418 is not a simple lysosomotropic agent. a Western blot of LAMP1 with UNI418 and Chloroquine. GAPDH was used as a loading control. NT, not treated. **b** Immunofluorescence images showing accumulation of LAMP1 in RPE1 cells with the indicated concentration of Chloroquine and UNI418 treatment, and images within the white rectangular boxes are zoomed in on the right side. Scale bar, 20 µm. **c** The mean of intensity in individual cell was quantified. Data presented as mean ± SEMs, n >20. Significance was determined by Student's t-test, ***$p < 0.001$.

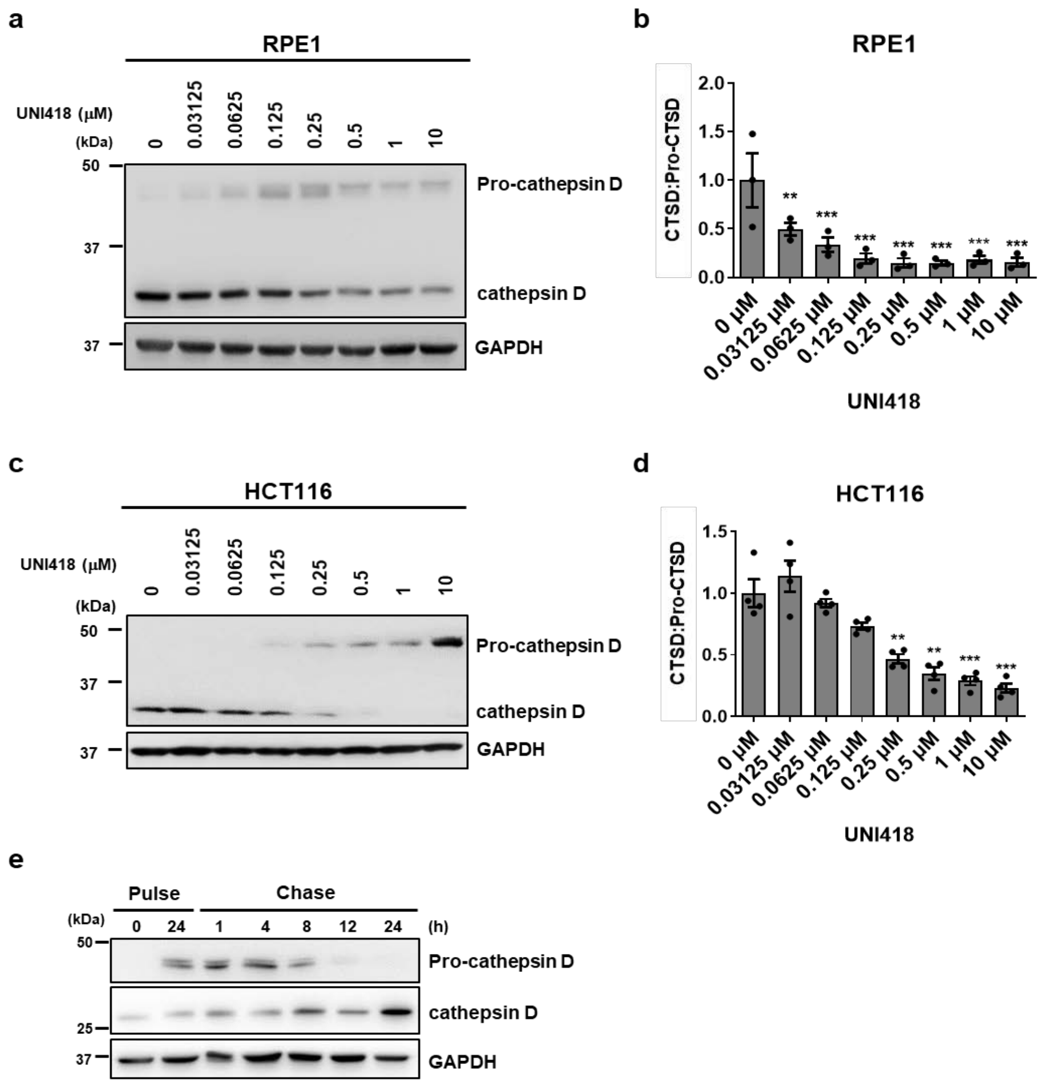

Supplementary Fig. 10 UNI418 inhibits the proteolytic maturation of cathepsin. a, b Western blot analysis showing inhibition of cathepsin D maturation by UNI418 in RPE1 cells and its quantification by measuring the band intensities of cathepsin D (CTSD) and its pro-form (Pro-CTSD). **c, d** Western blot analysis showing inhibition of cathepsin D maturation by UNI418 in HCT116 cells and its quantification by measuring the band intensities of CTSD and Pro-CTSD. **a, c** GAPDH was used as a loading control. **b, d** The ratios of CTSD to Pro-CTSD are presented as means ± SEM, n=4. Significance was determined by Student's t-test, **p* < 0.05, ***p* < 0.01 and ****p* < 0.001. **e** Western blot results of cathepsin D alteration at the each time point after UNI418 treatment or release after treatment.

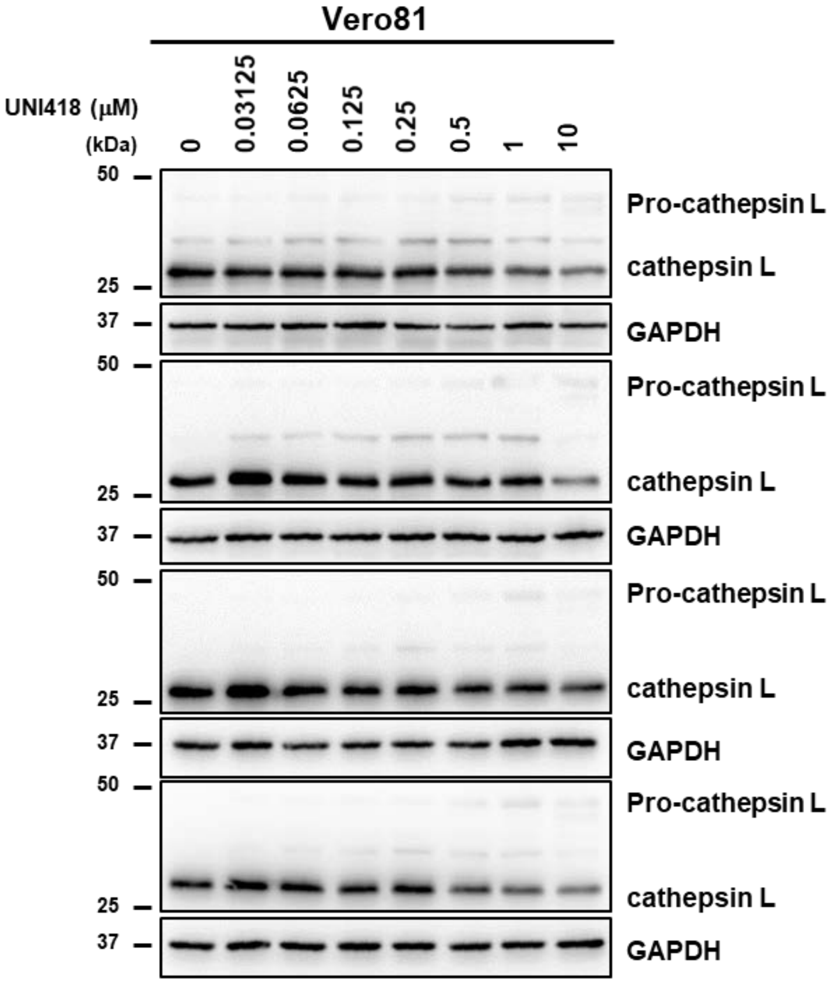

Supplementary Fig. 11 Western blot analysis of cathepsin L maturation in Fig. 4e.

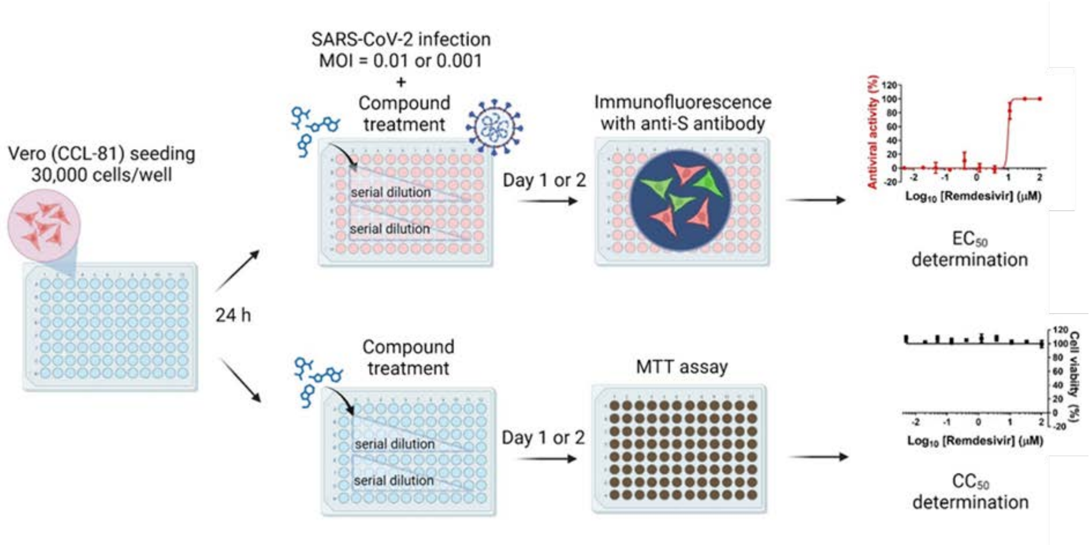

Supplementary Fig. 12 The workflow figure for antiviral assay and cytotoxicity test in Fig. 5a.

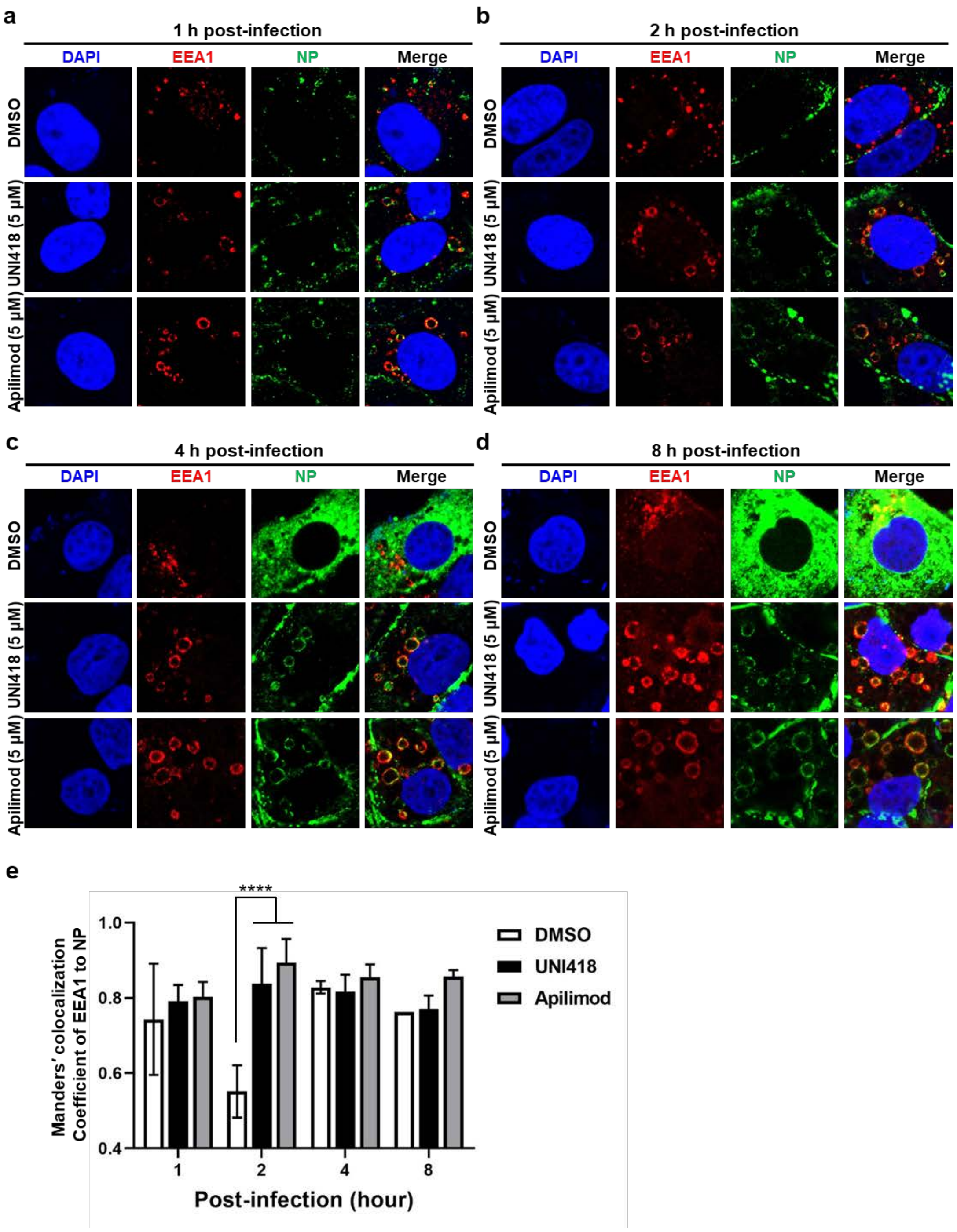

Supplementary Fig. 13 Immunofluorescence staining showing the viral NP (green) and cellular EEA1 (red) in SARS-CoV-2-infected Vero cells. a to **d** Separate and merged, zoomed-in images from Fig. 5d. **e** Manders' colocalization coefficient of EEA1 to NP. Statistical significance was determined using an unpaired t-test, ****$p < 0.0001$.

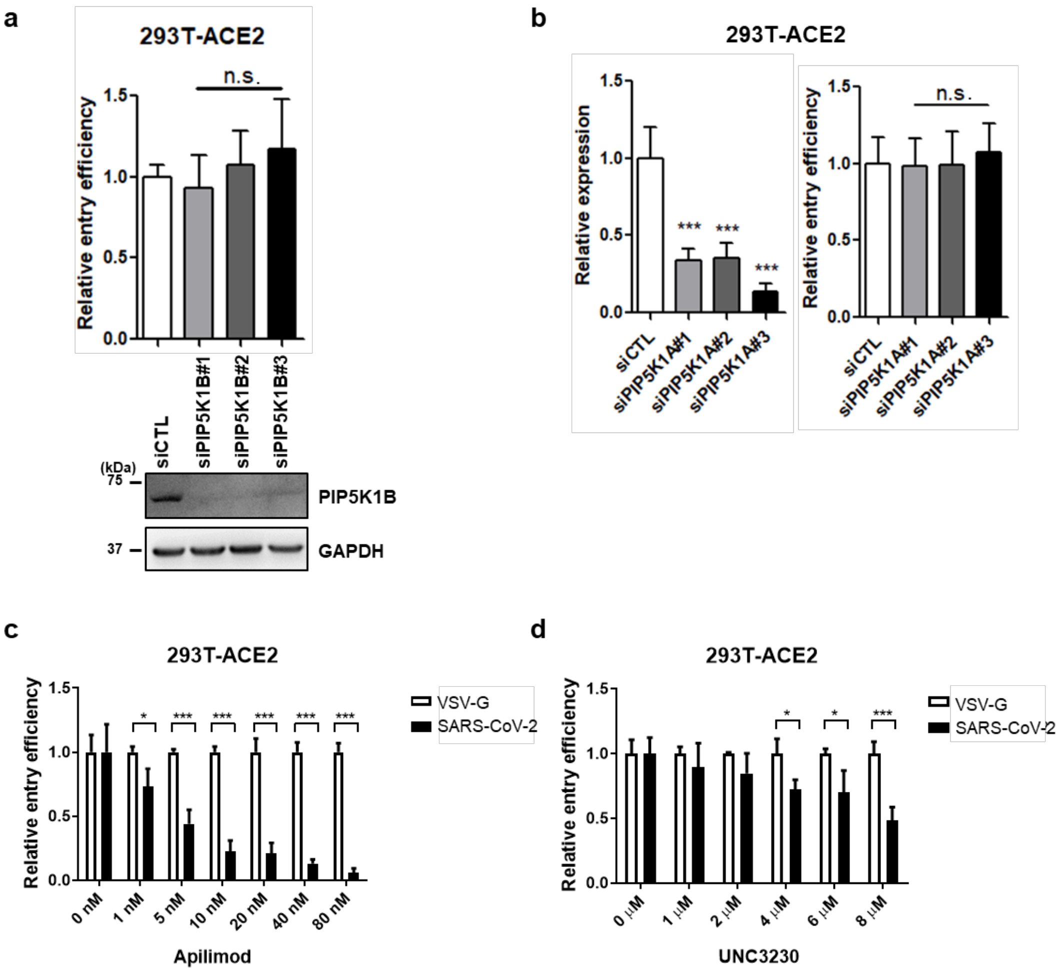

Supplementary Fig. 14 Viral entry assays of SARS-CoV-2 Spike-pseudotyped virus. a Virus entry of WuHan-Hu-1 Spike-pseudotyped was determined after PIP5K1B knockdown in 293T-ACE2 cells. HEK293T-ACE2 cells were transfected with indicated siRNAs and subjected to luciferase-based virus entry assay (top panel) and Western blot (bottom panel). **b** Pseudo-typed virus entry assay was analyzed after PIP5K1A knockdown in 293T-ACE2 cells and subjected to luciferase-based virus assay (right panel) and check expression of PIP5K1A using real-time PCR (left panel). **c, d** Pseudo-typed virus entry assay with Apilimod (**c**) and UNC3230 (**d**) in a dose dependent manner. Data presented as mean ± S.D, n=3. Significance was determined by Student's t-test, *p < 0.05, ***p < 0.001 and n.s., not significant.

Supplementary References

1. Lee JY, Koga H, Kawaguchi Y, Tang W, Wong E, Gao YS *et al.* HDAC6 controls autophagosome maturation essential for ubiquitin-selective quality-control autophagy. *EMBO J.* **29,** 969-980 (2010).
2. Klionsky DJ, Abdel-Aziz AK, Abdelfatah S, Abdellatif M, Abdoli A, Abel S *et al.* Guidelines for the use and interpretation of assays for monitoring autophagy (4th edition)(1). *Autophagy* **17,** 1-382 (2021).
